# Heterodimerization of Arabidopsis calcium/proton exchangers contributes to regulation of guard cell dynamics and plant defense responses

**DOI:** 10.1093/jxb/erx209

**Published:** 2017-06-22

**Authors:** Bradleigh Hocking, Simon J Conn, Murli Manohar, Bo Xu, Asmini Athman, Matthew A Stancombe, Alex R Webb, Kendal D Hirschi, Matthew Gilliham

**Affiliations:** 1 Waite Research Institute and School of Agriculture, Food and Wine, University of Adelaide, Glen Osmond, SA, Australia; 2 ARC Centre of Excellence in Plant Energy Biology, University of Adelaide, Glen Osmond, SA, Australia; 3 US Department of Agriculture/Agricultural Research Service, Children’s Nutrition Research Center, Baylor College of Medicine, Houston, TX, USA; 4 Department of Plant Sciences, University of Cambridge, Cambridge, UK

**Keywords:** Calcium, guard cells, homeostasis, mesophyll, protein interaction, signaling, transport

## Abstract

*Arabidopsis thaliana* cation exchangers (CAX1 and CAX3) are closely related tonoplast-localized calcium/proton (Ca^2+^/H^+^) antiporters that contribute to cellular Ca^2+^ homeostasis. CAX1 and CAX3 were previously shown to interact in yeast; however, the function of this complex in plants has remained elusive. Here, we demonstrate that expression of *CAX1* and *CAX3* occurs in guard cells. Additionally, *CAX1* and *CAX3* are co-expressed in mesophyll tissue in response to wounding or flg22 treatment, due to the induction of *CAX3* expression. Having shown that the transporters can be co-expressed in the same cells, we demonstrate that CAX1 and CAX3 can form homomeric and heteromeric complexes in plants. Consistent with the formation of a functional CAX1-CAX3 complex, *CAX1* and *CAX3* integrated into the yeast genome suppressed a Ca^2+^-hypersensitive phenotype of mutants defective in vacuolar Ca^2+^ transport, and demonstrated enzyme kinetics different from those of either CAX protein expressed by itself. We demonstrate that the interactions between CAX proteins contribute to the functioning of stomata, because stomata were more closed in *cax1-1*, *cax3-1*, and *cax1-1/cax3-1* loss-of-function mutants due to an inability to buffer Ca^2+^ effectively. We hypothesize that the formation of CAX1-CAX3 complexes may occur in the mesophyll to affect intracellular Ca^2+^ signaling during defense responses.

## Teaser

The cation exchanger family members CAX1 and CAX3 form homomeric and heteromeric complexes that are likely to modulate calcium transport during signaling events.

## Introduction

The tight control of calcium concentration ([Ca^2+^]) within the apoplast (cell wall) and symplast (cytosol, vacuole, and other endomembrane compartments) are critical for plant nutrition, structure, development, signaling, and physiology (Pittman and Hirschi, 2003; White and Broadley, 2003; Hetherington and Brownlee, 2004; Pittman *et al.*, 2005; Dodd *et al.*, 2010; Conn *et al.*, 2011*b*; Gilliham *et al.*, 2011). Transporters that reside in the plant vacuolar membrane (the tonoplast) play a major role in the regulation of [Ca^2+^] within the apoplastic and symplastic compartments (Pottosin and Schönknecht, 2007; Conn *et al.*, 2011*b*). The Ca^2+^/H^+^ antiporters CAX1 and CAX3 were previously identified as tonoplast-localized transporters that are important in controlling tissue Ca^2+^ homeostasis (Hirschi *et al.*, 1996; Shigaki *et al.*, 2001; Cheng *et al.*, 2005; Conn *et al.*, 2011*b*; Manohar *et al.*, 2011*a*, 2011*b*; Pittman and Hirschi, 2016). These proteins share 87% sequence similarity and 79% sequence identity, and function as low-affinity, high-capacity Ca^2+^ transporters that use the protomotive force generated by the vacuolar H^+^-ATPase and PPi-dependent H^+^ pumps to sequester Ca^2+^ from the cytosol into the vacuole (Hirschi *et al.*, 1996).

Both CAX1 and CAX3 proteins have been ascribed a functional role based on *in planta* expression analysis, ectopic expression, and mutant analysis in plants, and by heterologous expression in yeast (Shigaki and Hirschi, 2000; Manohar *et al.*, 2011*b*). Until now, *CAX1* and *CAX3* expression has been shown to overlap in reproductive tissues at the organ level, but to localize differentially within the vegetative organs. *CAX3* expression has been shown to localize primarily to root tips, whereas *CAX1* expression is predominantly localized to leaf tissues. CAX1 regulates elemental accumulation across specific leaf cell types and subcellular compartments (Hirschi *et al.*, 1996; Catalá *et al.*, 2003; Conn *et al.*, 2011*b*), whereas root growth in *cax3-1* plants is lower than that of wild-type or *cax1-1* plants under saline conditions, a phenotype that has been attributed to the greater inhibitory effects of Na^+^ (and Li^+^) on CAX3 Ca^2+^ transport compared with CAX1 (Cheng *et al.*, 2003; Manohar *et al.*, 2011*b*).

*CAX* expression in leaves appears to be variable and subject to unresolved regulatory mechanisms (Conn and Gilliham, 2010; Gilliham *et al.*, 2011). For example, in *cax1-1* plants, expression of *CAX3* and *CAX4* increase along with a vacuolar Ca^2+^-ATPase *ACA4* (Cheng *et al.*, 2003). It has been hypothesized that enhanced *CAX3* expression complements for the loss of *CAX1*, as *cax1-1* plants do not show the major physiological perturbations of *cax1-1/cax3-1* plants; however, it has not been shown whether *CAX3* directly replaces *CAX1* in the mesophyll (Cheng *et al.*, 2003; Conn *et al.*, 2011*b*). Such a complex system of cross-talk among genes is proposed to account for the subtleties in phenotypes that are often associated with loss-of-function genetic studies for transport proteins (Connorton *et al.*, 2012). In order to elucidate these compensatory changes, in the present study we designed experiments to analyze the potential interactions between CAX transporters.

Despite the existence of phenotypic differences between *cax* knockout plants, there are also notable similarities. For instance, germination in both *cax1-1* and *cax3-1* knockouts is abscisic acid (ABA) and sugar sensitive, and ethylene inhibits seedling growth of both mutants, which suggests some shared functionality of these proteins (Zhao *et al.*, 2008). Previous functional assays of CAX1 and CAX3 proteins in yeast, although insightful, have limitations because they used engineered CAX variants that lack N-terminal autoregulatory domains (Pittman and Hirschi, 2016). The functional significance of CAX1 and CAX3 interactions suggested by genetic and yeast assays is yet to be fully understood (Zhao *et al.*, 2009*b*). Such interactions may occur in the guard cell, as expression of both *CAX1* and *CAX3* has been detected in guard cell protoplasts (Leonhardt *et al.*, 2004; Cho *et al.*, 2012). Moreover, genetic analysis has implied that a putative CAX1-CAX3 complex may influence guard cell closure and apoplastic pH (Cho *et al.*, 2012).

Here, we provide evidence that heteromeric CAX complexes have physiological roles in plants. A split luciferase assay demonstrated that CAX1 and CAX3 form homo- and heterodimers in plant tissue, and, using yeast-based functional assays, we have shown for the first time that full-length CAX1-CAX3 has distinct transport characteristics compared with homomeric truncated-deregulated CAX proteins. The functional role for a CAX1-CAX3 complex in the plant was probed with stomatal assays, and both CAX1 and CAX3 appear to be required for correct functioning of the stomata. Our results highlight that expression of *CAX3* and, to a lesser extent, *CAX1* is induced in the mesophyll during defense responses, and that both proteins are required in the guard cell for the control of gas exchange. The interactions between CAX1 and CAX3 identified here suggest the possibility of regulatory plasticity in tonoplast Ca^2+^ transport during signaling events.

## Materials and methods

### Plant materials and growth

All chemicals were obtained from Sigma-Aldrich unless stated otherwise. Plant materials were *Arabidopsis thaliana* wild-type Columbia-0 (Col-0) and Col-0 background T-DNA insertional loss-of-function mutants *cax1-1*, *cax3-1*, and *cax1-1/cax3-1 (cax1/cax3*) (Cheng *et al.*, 2005). For soil growth, seeds were sown on zero-nutrient-containing coco-peat-based soil and supplied weekly with a defined basal nutrient solution (BNS: 2 mM NH_4_NO_3_, 3 mM KNO_3_, 0.1 mM CaCl_2_, 2 mM KCl, 2 mM Ca(NO_3_)_2_, 2 mM MgSO_4_, 0.6 mM KH_2_PO_4_, 1.5 mM NaCl, 50 µM NaFe(III)EDTA, 50 µM H_3_BO_3_, 5 µM MnCl_2_, 10 µM ZnSO_4_, 0.5 µM CuSO_4_, 0.1 µM Na_2_MoO_3_, adjusted to pH 5.6 by the addition of KOH) as previously described by Conn *et al.* (2013). Hydroponic growth also followed the method described by Conn *et al.* (2013), with the following exceptions. After root emergence from modified microcentrifuge tubes containing low-nitrate germination medium at ~2 weeks, these tubes were transferred to aerated hydroponics tanks containing either BNS (2 mM Ca^2+^) or 300 µM sufficient but low calcium solution (SLCS) for another 3 weeks before a further Ca^2+^ treatment in BNS, modified BNS using 11 mM high Ca^2+^ Solution (HCS), or SLCS; see Supplementary Table S1 at *JXB* online for full solution composition. All plants were grown in a short-day growth room (9.5 h light/15.5 h dark, 110 µmol m^−2^ s^−1^, 19 °C). Calcium concentration measurements were performed as previously described by Cheng *et al.* (2002).

### RNA extraction

Total RNA was extracted from shoot tissue or mesophyll protoplasts of 5–6-week-old Col-0 plants treated as indicated in the respective figure legends, using TRIzol reagent (Invitrogen) and the DNase-treated by Turbo DNA-free kit (Ambion). Reverse transcription was used to synthesize cDNA from 2 µg RNA from each sample using SuperScript^®^ III Reverse Transcriptase (Invitrogen) with Oligo(dT)_20_ as previously described by Conn *et al.* (2011*b*).

### Gene cloning and plasmid construction

PCR was used to amplify DNA fragments from Arabidopsis cDNA to clone the *CAX1* and *CAX3* coding sequences without start or stop codons (primers listed in Supplementary Table S2). Then, the DNA fragments were cloned into the pCR8/GW/TOPO TA cloning vector and transformed into TOP10 chemically competent *Escherichia coli* (Invitrogen). The genes of interest in pCR8/GW/TOPO vectors were recombined into serial pDuEx-Bait/Prey expression vectors for a split luciferase interaction assay (Nakagawa *et al.*, 2007), and a subsequent Cre-Lox recombinase reaction was performed to produce dual gene expression vectors for simultaneous expression of NLuc-*CAX1* and *CAX3*-CLuc (or CLucN-*CAX3*-CLuc) (Creator^TM^ DNA Cloning Kit, Clontech). *CAX1* and *CAX3* promoters (2 kb region upstream of the gene start codon ATG) as described by Cheng *et al.* (2005) were amplified from *A. thaliana* (Col-0) genomic DNA. Primers incorporated *Eco*RI/*Hin*dIII restriction sites (Supplementary Table S2) with the amplicon subcloned into pNO::Luc vectors using T4 DNA Ligase (New England Biolabs). The *CAX1* and *CAX3* artificial miRNA (amiRNA) was designed to achieve *CAX*-specific transcript reduction. *CAX1* and *CAX3* amiRNA sequences were designed using Web MicroRNA Designer v2 (Schwab *et al.*, 2006) and cloned into the pCR8/GW/TOPO vector. Subsequently, *CAX1* and *CAX3* amiRNA was recombined into a 2× CaMV 35S overexpression vector (pTOOL2) and used for miRNA expression (Plett *et al.*, 2010).

### Semi-quantitative PCR

Semi-quantitative PCR (semi-qPCR) for cell-specific *CAX1* and *CAX3* expression analysis was performed on cDNA separately synthesized from RNA of 5–8-week-old Arabidopsis mesophyll and epidermal cells. RNA preparation was performed as described previously using single cell sampling (SiCSA) (Conn *et al.*, 2011*b*). Transcripts amplified were *Actin2* (At3g18780; normalization control for both epidermis and mesophyll), *CAX1* (At2g38170), and *CAX3* (At3g51860); primers used are listed in Supplementary Table S2. Amplification for this analysis was performed using Phire Hot Start Taq DNA Polymerase (Finnzymes) with the following cycling conditions: first round: 98 °C for 1 min, then 25 cycles of 98 °C for 10 s, 50 °C for 10 s, and 72 °C for 30 s. Primers were then removed using Nucleospin Extract II (Macherey-Nagel), and 1 μl of the eluate was used as the template for the second round: 98 °C for 1 min, then 25 cycles of 98 °C for 10 s, 55 °C for 10 s, and 72 °C for 10 s.

Semi-qPCR for *CAX1* and *CAX3* expression analysis was performed on cDNA samples reverse-transcribed from mesophyll protoplast RNA isolated from 5–6 week-old Arabidopsis leaves, following the protoplast isolation method detailed below. Transcripts amplified were *Actin2*, *CAX1*, *CAX3*, and *GC1* (At1g22690; a marker gene for guard cells) with primers listed in Supplementary Table S2. Amplification of *Actin2*, *CAX1*, *CAX3*, and *GC1* transcripts was performed using Phire Hot Start DNA Polymerase (Finnzymes) with the following cycling conditions: 98 °C for 1 min; 35 cycles of 98 °C for 5 s, 56 °C for 5 s, and 72 °C for 15 s; and then 72 °C for 1 min.

### *In situ* PCR

Leaves of 5–6-week-old Arabidopsis plants grown in short-day conditions were infiltrated with either ultrapure H_2_O or 2 µM flg22 in ultrapure H_2_O and left for 12 h. Then, leaves were detached, cut into 5 mm strips, and fixed in ice-cold formalin-acetic-alcohol solution [63% (v/v) ethanol, 5% (v/v) acetic acid, 2% (v/v) formalin] and washed in 1× PBS before being embedded in 5% agarose. Embedded leaf tissue was cross-sectioned using a VT 1200 S Vibrating Microtome (Leica) into 70 μm sections and transferred into a PCR tube. Then, the *in situ* PCR protocol of Athman *et al.* (2014) was followed using gene-specific qPCR primers as listed in Supplementary Table S2, with the following cycling conditions: 98 °C for 1 min; 35 cycles of 98 °C for 5 s, 56 °C for 5 s, and 72 °C for 15 s; and then 72 °C for 1 min.

### Real-time quantitative PCR

Real-time quantitative PCR (RT-qPCR) was performed on 0.2 µl cDNA using an iCycler Thermal cycler equipped with an iQ multi-color optical assembly module (Bio-Rad) and using KAPA SYBR^®^ FAST qPCR Kits (KAPA Biosystem), according to the following program: 95°C for 3 min; 40 cycles of 95 °C for 20 s, 55 °C for 20 s, and 72 °C for 20 s; with melt curve analysis from 52 °C to 92 °C in 0.5 °C increments. Primers for RT-qPCR analysis are listed in Supplementary Table S2. RT-qPCR result analysis followed the method described by Schmittgen and Livak (2008) using 2^−Δ*C*т^ to calculate gene expression level normalized to *Actin2* (At3g18780) as an internal control.

### Protoplast isolation

**Fig. 1.**
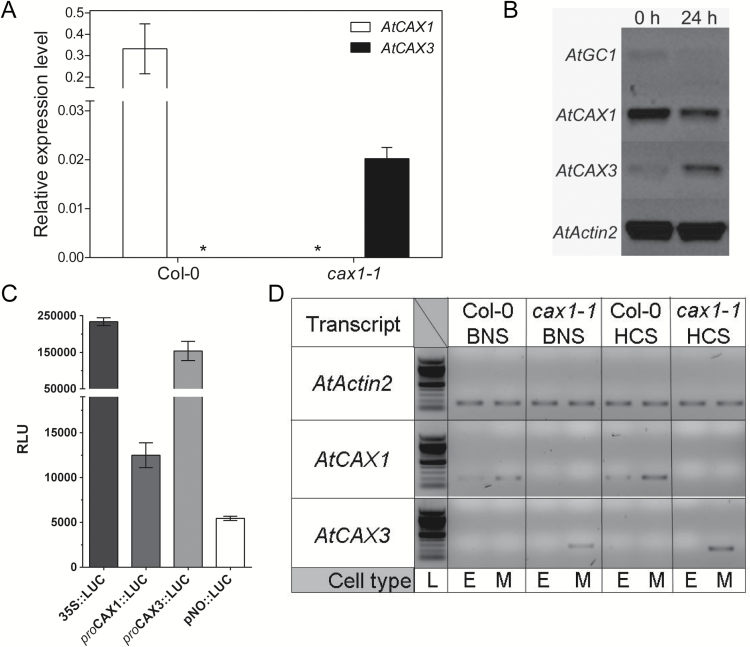
Profiling *CAX1* and *CAX3* transcript expression and promoter activity in leaf tissue and protoplasts. (A) Laser capture microdissection and qPCR of Col-0 and *cax1-1* leaf mesophyll cells. Data represent mean±SD, *n*=3 plants, performed in triple technical replicates. Gene transcript level was normalized to *Actin2* (At3g18780). Asterisks indicate undetectable transcript level. (B) Semi-qPCR of Col-0 mesophyll protoplasts after 0 or 24 h in protoplast culture. (C) Expression of *CAX* native promoter (full length and fragments)/luciferase fusions in Col-0 mesophyll protoplasts. (D) SiCSA and semi-qPCR of Col-0 and *cax1-1* grown in basal nutrient solution (BNS) and high Ca^2+^ solution (HCS).

Isolation and transformation of protoplasts was carried out according to a previously described method (Yoo *et al.*, 2007). Briefly, mesophyll protoplasts were isolated from leaf strips of 5–6-week-old *A. thaliana* by a 3-hour digestion in an enzyme solution containing 1.5% cellulase R10 and 0.4% macerozyme R10 (Yakult Pharmaceutical). Protoplasts were transformed via the polyethylene glycol 1450-mediated introduction of plasmid DNA in buffer solution. Modifications to this method included the use of one cell incubation medium, W2 [4 mM 2-(*N*-morpholino)ethanesulfonic acid (MES), 0.4 M mannitol, 15 mM KCl, 10 mM CaCl_2_, and 5 mM MgCl_2_, adjusted to pH 5.7 with KOH], as a replacement for both WI and W5 solutions. Protoplasts were incubated at room temperature for 0 or 24 h before harvesting for RNA extraction as indicated in the legend of Fig. 1.

### Split luciferase protein–protein interaction and native promoter luciferase report assay

Direct protein–protein interactions between CAX1 and CAX3 were probed using a split luciferase complementation assay (Fujikawa and Kato, 2007). Mesophyll protoplasts were transformed with an equal amount of expression pDuEx-Bait and/or pDuEx-Prey plasmids and incubated for at least 16 hours. Native promoter reporter assays were performed using an equal amount of pNO::Luc expression plasmid fused to either the *35S*, *CAX1*, or *CAX3* promoter transformed into mesophyll protoplasts; pNO::Luc containing the promoter-free *LUC* gene was used as a negative control. Luciferase activity in the protein–protein interaction and promoter activity assay was detected using the ViviRen Live Cell Substrate (Promega) in a Polarstar Optima plate-reading spectrophotometer with luminescence detection capabilities (BMG Labtech). Following overnight incubation of transfected protoplasts, the ViviRen substrate was dissolved in DMSO and added to 500 µl protoplasts (2 × 10^5^ cells ml^−1^) to 60 µM, mixed briefly, and aliquots of 100 µl (equivalent to 4 × 10^4^ cells) were dispensed into white 96-well plates, in triplicate. Luminescence was measured immediately. Peak luminescence was observed at 300 s after substrate addition (gain=4095), and data from this time point were used for further analysis.

### Ca^2+^ tolerance and uptake assay in *Saccharomyces cerevisiae*

*Saccharomyces cerevisiae* strain K667 (*vcx1*::*hisG cnb1*::*LEU2 pmc1*::*TRP1 ade2-1 can1-100 his3-11*,*15 leu2-3,112 trp1-1 ura3-1*) (Cunningham and Fink, 1996) was transformed with *sCAX1* or *CAX1* and *CAX3* using (SC-Ura) transformation (Sherman *et al.*, 1986). The Ca^2+^ growth tolerance assay of *S. cerevisiae* was performed as previously described by Manohar *et al.* (2011*b*). Briefly, the assay was carried out via growing yeast expressing genes of interest at 30 °C for 3 days on solid YPD medium and supplemented with the appropriate amount of CaCl_2_. A vacuolar-enriched membrane fraction was prepared from yeast, following the method described by Manohar *et al.* (2011*b*). Yeast cells were collected by centrifugation at 4000× *g* for 5 min until the density reached an OD_600_ of ~1.5. The collected cell pellet was washed in spheroplast buffer (100 mM potassium phosphate buffer, 1.2 M sorbitol, pH 7.0) and resuspended in the same buffer plus 10 mM dithiothreitol (DTT) and 1% dextrose. Membrane vesicles of yeast cells were isolated using 1.5 units of zymolyase and incubated at 30 °C for up to 2 h. Time-dependent ^45^Ca^2+^/H^+^ transport into these endomembrane vesicles was measured as described previously by Pittman *et al.* (2005).

### Western blotting analysis

Western blotting analysis was performed as previously described by Manohar *et al.* (2011*b*). A monoclonal antibody to human influenza hemagglutinin (HA) (Berkeley Antibody Co., Richmond, CA, USA) was used at a 1:1000 dilution.

### Gas exchange and photosynthesis measurements

Gas exchange and photosynthesis rate of whole rosettes were measured in 5- to 8-week-old Arabidopsis plants with treatments as indicated in the corresponding figure legends, using a LI-6400 infrared gas exchange analyzer (LiCOR) equipped with an Arabidopsis whole-plant chamber. Individual plants were exposed to light intensity of ~350 μmol m^−2^ s^−1^ at least 30 minutes prior to the start of measurement. The rosette was allowed to acclimatize inside the Arabidopsis whole-plant chamber for at least 5 minutes before gas exchange data were recorded, with reference CO_2_ concentration set at 500 μmol mol^−1^, flow rate at 500 μmol s^−1^, light intensity at 350 μmol m^−2^ s^−1^, and relative humidity at 56%. Leaf area of the whole plant was calculated using MATLAB on the basis of the image of whole plants, as described by Conn *et al.* (2013).

### Guard cell aperture measurement

Stomatal aperture was measured in wild-type Col-0, *cax1-1*, *cax3-1*, and *cax1/cax3* lines, as described by Conn *et al.* (2011*b*). Briefly, epidermal peels were prepared from 3–4-week-old seedlings, grown on 0.5× Murashige and Skoog medium, and incubated in buffer containing 5 mM KCl, 10 mM MES-KOH (pH 6.2) with or without 1 mM CaCl_2_ supplement.

### *Agrobacterium*-mediated transformation

*Agrobacterium*-mediated Arabidopsis seedling transformation followed the Fast *Agrobacterium*-mediated Seedling Transformation (FAST) method described by Li *et al.* (2009). Briefly, Arabidopsis seedlings were grown on 0.25× Murashige and Skoog medium for 5–6 weeks before being transferred into fresh 0.25× Murashige and Skoog liquid medium in a Petri dish containing an additional 100 μM acetosyringone and 0.005% (v/v) Silwet L-77, and co-cultivation with *Agrobacterium tumefaciens* cells at OD_600_=0.5 for 2 days. Assays were then performed on these seedlings.

### Data and statistical analysis

Statistical tests are described in the figure legends. All graphing and statistical analysis were performed in GraphPad Prism v6 and 7.

## Results

### *CAX3* expression can be induced in mesophyll cells by *cax1* knockout, wounding, or pathogen stress

To directly test whether it is possible for *CAX3* to replace and compensate for the loss of *CAX1* expression in *cax1-1* plants, we examined the expression profile of *CAX1* and *CAX3* in mesophyll cells of *A. thaliana* ecotype Col-0 and *cax1-1* using laser capture microdissection (LMD) qRT-PCR (Fig. 1A). We confirmed that *CAX1* was expressed at high levels in the mesophyll cells of wild-type (Col-0) plants, whereas *CAX3* was not detected. In contrast, *CAX3* expression was significantly induced in *cax1-1* mesophyll, when *CAX1* was absent, as predicted by Conn *et al.* (2011*b*). This suggests that it is possible for *CAX3* to be expressed in the mesophyll under some conditions. However, the lack of *CAX3* expression detected in Col-0 mesophyll cells (Fig. 1A), contrasts with previous observations made from protoplasts, where both *CAX1* and *CAX3* have been detected (Leonhardt *et al.*, 2004; Cho *et al.*, 2012). We further explored this disparity.

Immediately following the isolation of mesophyll protoplasts, *CAX3* expression was barely detected, but the expression was significantly increased 24 h after protoplast isolation. Interestingly, *CAX1* was highly abundant at both stages, with reduced expression after 24 h (Fig. 1B). A guard-cell-specific marker (GC1) (Yang *et al.*, 2008) was close to the detection limits at both time points (Fig. 1B). These results indicate that there was no or minimal guard cell contamination in our mesophyll preparation, and that *CAX3,* but not *CAX1,* showed inducible expression within mesophyll cells during the protoplasting procedure.

To assess whether these changes were due to either the stability of the mRNA or an increase in *CAX* promoter activity, we generated native *CAX* promoter::luciferase reporter constructs for transient expression in mesophyll protoplasts. To make this construct, we cloned the *CAX1* and *CAX3* promoter fragments, identified in Cheng *et al.* (2005) as reporting native expression patterns. We then inserted a promoter upstream of a luciferase protein derived from *Renilla reniformis* (sea pansy), and transfected the constructs into Arabidopsis mesophyll protoplasts. At 24 h after transfection, equivalent to the time point used in Fig. 1B, we could detect the expression of luciferase in the protoplasts driven by either the *CAX1* or the *CAX3* promoter (Fig. 1C), or via three sets of a truncated promoter for each CAX (F1, F2, and F3) (see Supplementary Fig. S1). Interestingly, the *CAX3* promoter drove 3- to 15-fold stronger luciferase activity compared with the respective *CAX1* promoter (Fig. 1C). This suggests that gene transcription of *CAX3* increases during protoplasting.

We then investigated whether we could induce *CAX3* expression in the mesophyll of Col-0 plants under any conditions. Using SiCSA and semi-qRT-PCR, under our standard conditions (i.e. growth in BNS), *CAX1* transcript was detected in both adaxial epidermal and palisade mesophyll cells of Col-0 leaves, whereas *CAX3* was detected only from *cax1-1* plant mesophyll (Fig. 1D). *CAX1* transcript was abundant in RNA extracted from whole leaves of Col-0 plants, consistent with its expression in epidermis and mesophyll, while the presence of *CAX3* transcript was below the level of our assay’s detection limits under our standard growth conditions (see Supplementary Fig. S2). In an attempt to overload the leaf with apoplastic Ca^2+^, to mimic the situation in *cax1/cax3* plants (Conn *et al.*, 2011*b*), we increased the concentration of Ca^2+^ in the root growth solution from 2 to 11 mM Ca^2+^ (HCS). Although both transcripts were induced by HCS (Fig. 1D; Supplementary Fig. S2), we did not observe a change in the cell-type localization of *CAX1* and *CAX3* expression under high Ca^2+^ conditions (Fig. 1D). Furthermore, *w*e compared the leaf vacuolar and apoplastic Ca^2+^ content of Col-0, *cax1-1*, and *cax3-1*, and no significant differences were observed between the genotypes (Supplementary Fig. S3).

**Fig. 2.**
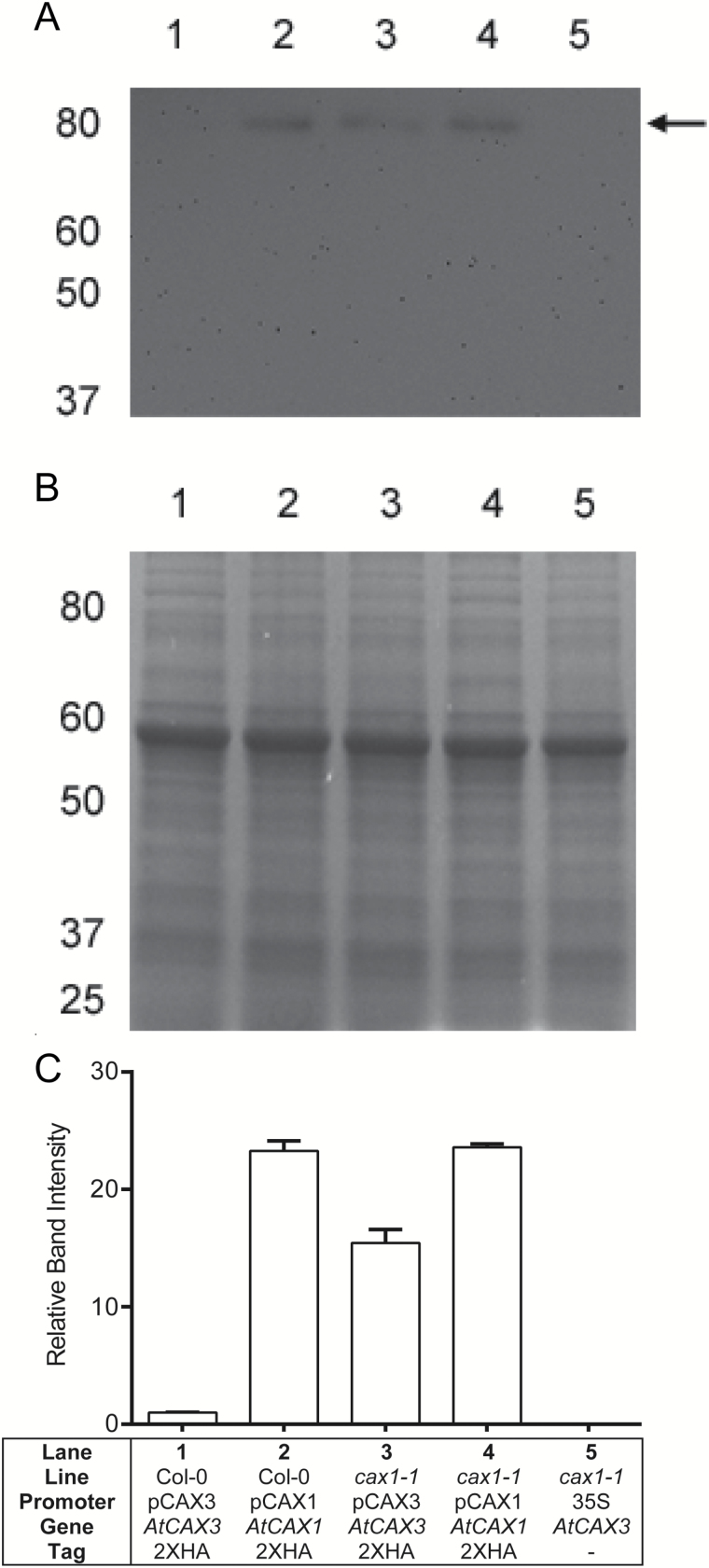
Levels of CAX1 and CAX3 protein expression in Col-0 and *cax1-1* plants as assessed using the FAST technique (Li *et al.*, 2009). The following native promoter/gene/reporter fusions were constructed: pCAX1::AtCAX1::YFP::2xHA (pSIM1; GenBank accession number: HM750245.1) and pCAX3::AtCAX3::YFP::2xHA (pSIM3; GenBank accession number: HM750246.1). pSIM1 and pSIM3 proteins were predicted to have molecular weights of 81.9 and 81.6 kDa, respectively (ExPASy Compute pI/Mw tool). Arabidopsis Col-0 seedlings were co-cultivated with *Agrobacterium* carrying (1) pSIM3, (2) pSIM1; the *cax1-1* line was transformed with (3) pSIM3 and (4) pSIM1; and (5) d35S::AtCAX3 served as a negative control. (A) Western blot of total protein extracted from the shoots of eight transformed seedlings, transferred to nitrocellulose membrane, and probed with anti-HA, HRP-conjugated primary antisera (Cell Signalling Technology, CS2999), demonstrates that *CAX3* compensates at the protein level for the loss of *CAX1* in the *A. thaliana cax1-1* T-DNA insertion line. (B) Coomassie-stained 10% SDS-PAGE gel as a loading control for the Western blot. (C) Quantification of band intensities across three biological replicates using QuantityOne software (Bio-Rad Laboratories), normalized to pSIM3-transfected Col-0 (lane 1).

To determine whether the changes in *CAX* transcript abundance translated into increases in CAX protein levels, we used immunochemistry to measure the abundance of translational pCAX(1 or 3):CAX::HA fusions. In Col-0, the relative amount of CAX1 and CAX3 protein reflected the differences in transcript abundance, with CAX1 ~24-fold as abundant as CAX3 (Fig. 2). In *cax1-1* plants transformed with these constructs, CAX1 protein abundance was comparable to wild-type, whereas CAX3 protein abundance was increased ~17-fold compared with wild-type (Fig. 2). These results strongly indicate that the changes in transcript abundance for *CAX1* and *CAX3* in whole leaves and in protoplasts reflect changes in protein abundance. This finding encouraged us to conduct a broader analysis to determine whether there were other stimuli that alter *CAX* expression.

**Fig. 3.**
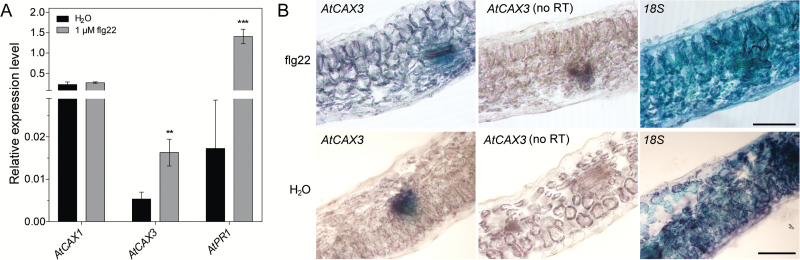
*CAX3* expression in leaves increases upon flg22 treatment. (A) Expression level of *CAX1*, *CAX3*, and *PR1* in shoots of Arabidopsis Col-0 12 h after leaf infiltration of 1 μM flg22. Data represent mean±SD, *n*=3 plants, performed in triple technical replicates. Gene transcript level was normalized to *Actin2* (At3g18780). Statistical analysis as determined by Student’s *t* test: **P<0.01, ***P<0.001. (B) *In situ* PCR of *CAX3* expression in wild-type leaf cross sections. 5–6-week-old Arabidopsis leaves were infiltrated with either H_2_O or 1 µM flg22 for 12 h before fixation. *CAX3* and *18S* rRNA transcripts were amplified with primers as listed in Supplementary Table S1 before staining; *18S* rRNA was used as a positive control to show the presence of cDNA in all cell types; a no reverse transcription (RT) control was included to show lack of genomic DNA contamination. Scale bars=100 µm.

A survey of existing Arabidopsis expression data (eFP BAR; http://bbc.botany.utoronto.ca) identified several biotic and abiotic stresses that might regulate *CAX1* and *CAX3* expression. *CAX3* abundance was increased in leaves/shoots by osmotic stress, salt stress, and infection with *Pseudomonas syringae* or *Botrytis cinerea* (Table 1). To further examine the effect of *P. syringae* on *CAX* expression, we infiltrated 5-week-old Col-0 leaves with 1 μM flg22 (as a proxy for *Pseudomonas* infection) or water (as a control) 12 h before performing qPCR. Consistent with the result in the eFP BAR database, *CAX3* expression was increased in flg22-infiltrated leaves, whereas *CAX1* expression was unchanged (Fig. 3A). In addition, using *in situ* PCR, *CAX3* was detected in the mesophyll of flg22-infiltrated leaves but not in the leaves of water-infiltrated controls (Fig. 3B). Protoplasting induces wound responses (Ecker and Davis, 1987) and flg22 is a pathogen mimetic; both induce *CAX3* expression, which implies that CAX3 has a role in plant defense responses. Using the BAR Expression Angler, we identified 236 genes strongly co-regulated with *CAX3* (*r*^2^>0.75) in Arabidopsis leaves in response to *P. syringae*, *B. cinerea*, and their corresponding elicitors, whereas there were only 16 genes co-regulated in response to abiotic stresses (Austin *et al.*, 2016). Furthermore, microarray analysis of *cax1/cax3* plants shows alteration in expression of many pathogen-related genes (see Supplementary Table S3). These findings suggest that co-expression of *CAX1* and *CAX3* within the mesophyll occurs during defense responses and as such there is potential for a CAX1-CAX3 complex to have a physiological role.

**Table 1.** *In silico analysis of* CAX1 *and* CAX3 *expression in Col-0 under various treatments*

Treatment	**Time after treatment (h**)	**Tissue**	***CAX1*** **(fold change)**	***CAX3*** **(fold change)**
Osmotic stress (300 mM mannitol)	24	Leaf	0.45	14.33
Salt (150 mM NaCl)	24	Leaf	0.71	4.57
		Root	2.06	5.98
Wounding (needle stick)	24	Leaf	1.07	2.86
*Pseudomonas syringae* infiltration	2, 6, 24	Leaf	0.73, 0.83 0.9	1.04, 0.56, 7.91
1 μM flg22 infiltration	1, 4	Leaf	0.62, 0.43	0.96, 0.77
*Botrytis cinerea*	18, 48	Leaf	1.0, 1.27	7.74, 11.68
50 μM ABA	3	Leaf	1.22	5.18
		Guard cells from epidermal peels	0.24	5.69
100 μM ABA	4	Mesophyll protoplasts	0.57	1.14
		Guard cell protoplasts	1.04	2.59
10 μM ABA	3	7-day-old seedlings	0.82	5.38

This table includes expression data from a variety of experiments; as such, conditions are not standardized between experiments. Data are expressed as fold change compared with mock-treated tissue. ABA, abscisic acid. Adapted from eFP BAR; http://bbc.botany.utoronto.ca.

### CAX3 in guard cells affects stomatal responses

**Fig. 4.**
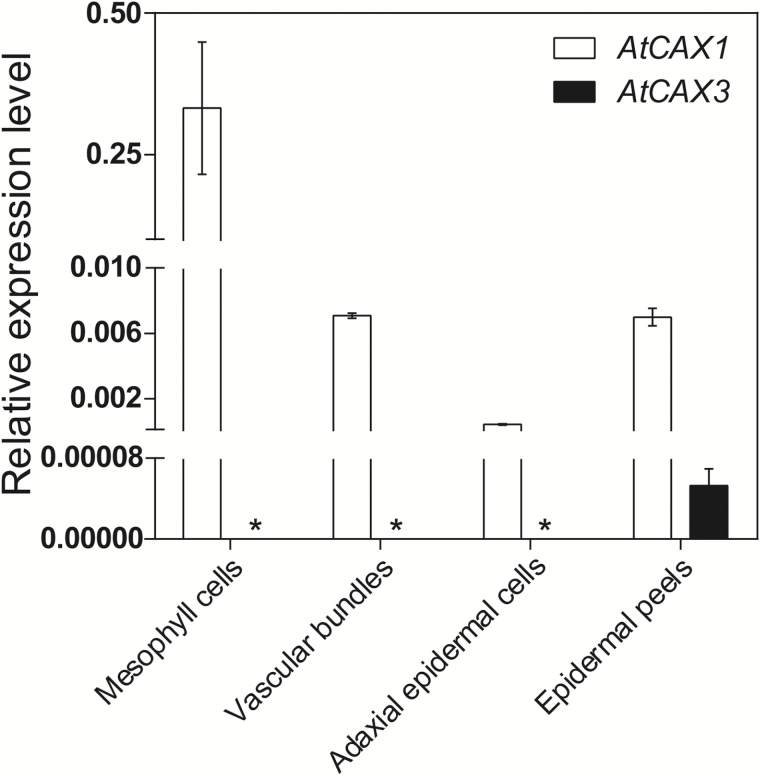
Expression profiling of *CAX1* and *CAX3* in leaf tissues. qPCR analysis of cDNA isolated from laser capture microdissected leaf cell types and tissues (leaf 8 of 6-week-old plants) of Col-0 Arabidopsis plants. Data represent mean±SD, *n*=3 plants, performed in triple technical replicates. Gene transcript level was normalized to *Actin2* (At3g18780). Asterisks indicate undetectable transcript level.

To determine whether the putative CAX1-CAX3 interaction may have functional roles in plants, we surveyed the cellular expression of *CAX1* and *CAX3* in leaves. *CAX1* expression was detected in mesophyll cells, vascular bundles, adaxial epidermal cells, and abaxial epidermal peels (Fig. 4). The only tissue assayed in which *CAX3* expression was detected was abaxial epidermal peels; these peels will contain viable stomatal guard cells, indicating that *CAX1* and *CAX3* are co-expressed in guard cells.

**Fig. 5.**
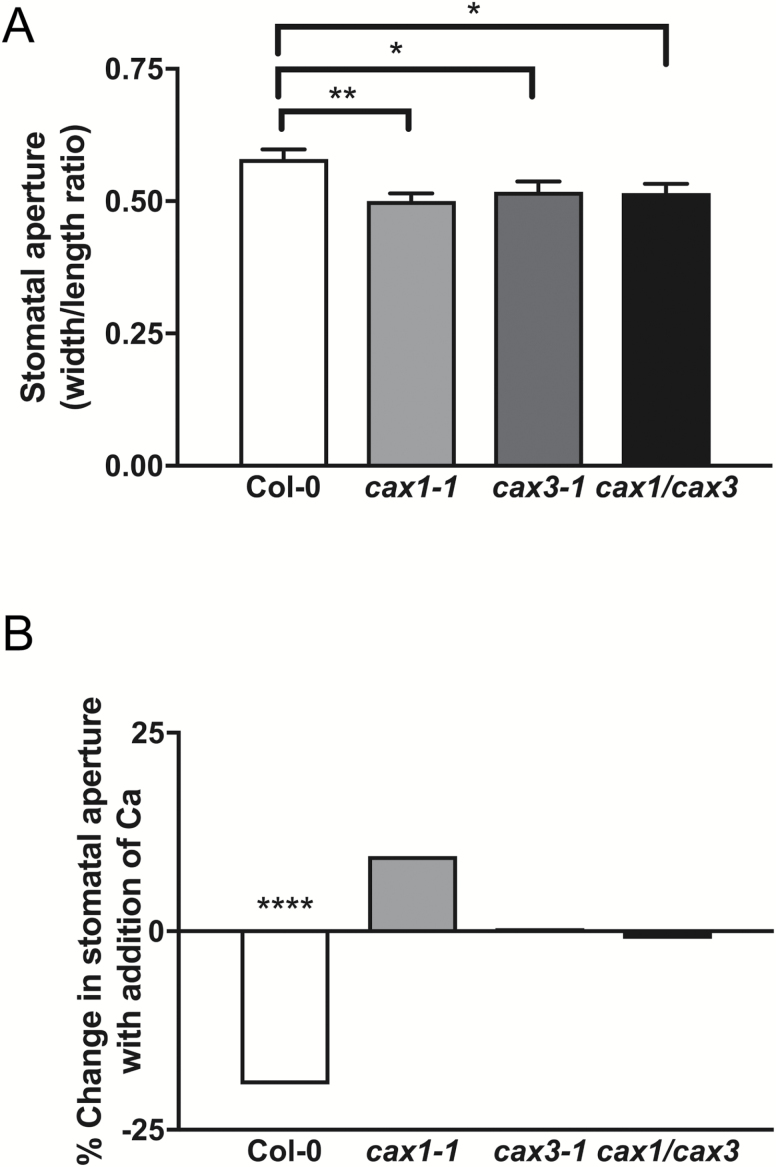
Stomatal aperture and calcium responsiveness of Col-0, *cax1-1*, *cax3-1*, and *cax1/cax3* isolated epidermal strips. (A) Stomatal pore aperture, expressed as width/length ratio, in Col-0, *cax1-1*, *cax3-1*, and *cax1*/*cax3*, *n*=175, 173, 173, 176, respectively. (B) Change in stomatal pore apertures in adaxial epidermal peels of Col-0, *cax1-1*, *cax3-1*, and *cax1/cax3* measured with an additional 1 mM extracellular Ca^2+^ (*n*=173, 183, 146, 166), plotted as the change in aperture between each genotype with or without the addition of 1 mM Ca^2+^. Asterisks indicate significant differences within a genotype with Ca^2+^ treatment: (A) two-way ANOVA with Tukey’s post-hoc test, **P=0.0054, *P<0.05; (B) Sidak’s multiple comparisons test, ****P<0.0001.

To investigate whether co-expression is required *in planta* for normal guard cell function, we examined the apertures of stomatal pores in Col-0, *cax1-1*, *cax3-1*, and *cax1/cax3* plants. Apertures of *cax1-1*, *cax3-1*, and *cax1/cax3* stomata from isolated epidermis were smaller than those of Col-0 (Fig. 5A). Moreover, none of the mutants had reduced apertures in the presence of supplemental extracellular [Ca^2+^], unlike Col-0 (Fig. 5B). Coupled with the fact that EGTA treatment opens *cax1/cax3* stomata (Conn *et al.*, 2011*b*), this result indicates that stomata in these epidermal peels were already partially closed in a Ca^2+^-dependent manner in both the single and double *cax* mutants. We previously demonstrated that while *cax1*/*cax3* exhibits reduced leaf gas exchange as a result of higher apoplastic calcium, which causes reduced stomatal aperture (Conn *et al.*, 2011*b*), apoplastic Ca^2+^ concentration was not different from wild type in *cax1-1* or *cax3-1* plants under steady-state conditions (see Supplementary Fig. 3). This is likely due to CAX1 protein, or CAX3 in the case of *cax1-1* plants, effectively buffering apoplastic Ca^2+^ in the mesophyll of the single-knockout plants.

**Fig. 6.**
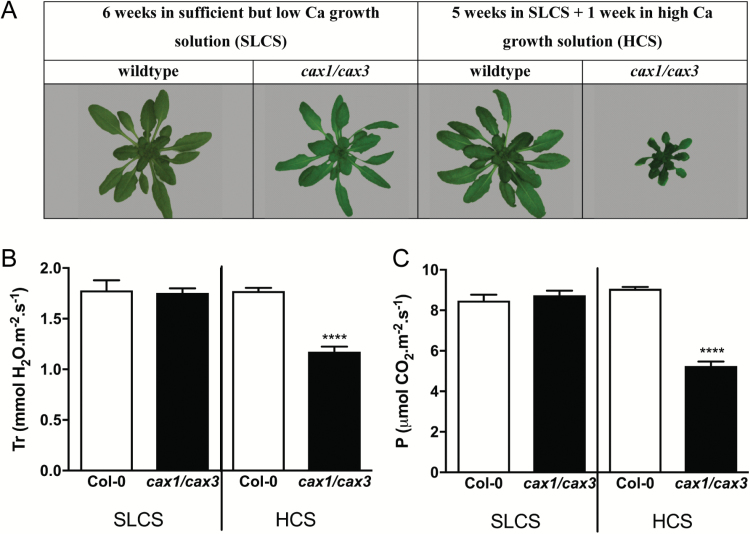
Growth and gas exchange phenotypes of Col-0 and *cax1/cax3* Arabidopsis under different Ca^2+^ nutrition regimes. (A) Example images showing the typical rosette size of Col-0 and *cax1/cax3* grown for 6 weeks in sufficient but low calcium solution (SLCS) or grown for 5 weeks in SLCS followed by 1 week in high calcium solution (HCS). Transpiration (B) and photosynthesis (C) rates were determined using a LI-6400 infrared gas exchange analyzer (LiCOR) equipped with an Arabidopsis whole-plant chamber, set up according to Conn *et al.* (2011*b*). Data are presented as mean±SEM, *n*=20. ****P<0.0001 (two-way ANOVA).

We therefore explored whether the Ca^2+^-sensitive stomatal phenotype of *cax1-1* and *cax3-1* plants could be recreated in leaves of intact plants or whether it was an artifact of the epidermal peel system. In order to achieve this, we first optimized the growth of *cax1/cax3* plants to ensure that the guard cell phenotype observed in intact *cax1/cax3* plants was not a consequence of the dwarf stature or delayed development of these plants when they are grown in standard BNS (2 mM Ca^2+^) growth solution (Conn *et al.*, 2011*b*). Previously, growing *cax1/cax3* plants in low Ca^2+^ solution (LCS, 50 μM Ca^2+^) mitigated growth inhibition and increased stomatal conductance compared with growth in BNS (Conn *et al.*, 2011*b*) (see Supplementary Fig. 4). However, at this low level of supplied Ca^2+^, plants developed necrotic lesions after 7 days. To overcome Ca^2+^ deficiency and optimize growth, we germinated and grew *cax1/cax3* plants in an optimized solution with low but sufficient calcium to support growth comparable to that of Col-0 (SLCS, 300 μM Ca^2+^) (Fig. 6). After 5 weeks we transferred plants from SLCS to high calcium solution (HCS, 11 mM Ca^2+^) for 1 week. While Col-0 plants did not appear to be adversely affected by this treatment and continued to develop normally, the growth of the *cax1/cax3* rosette was greatly inhibited (Fig. 6A). In addition, the photosynthesis and transpiration rate of *cax1/cax3* plants (per mm rosette surface area) was lower than that of Col-0 (Fig. 6B, C). Plants grown in SLCS for 6 weeks were used to compare the effects of root-fed Ca^2+^ on gas exchange as a proxy for stomatal aperture. Both photosynthesis and rosette conductance of the single *cax* knockout and wild-type lines were not significantly different in SLCS, but were decreased in the *cax1/cax3* line treated with HCS for 18 hours (Supplementary Fig. 5); this difference was sustained 7 days after treatment (data not shown). We found a decrease in mean rosette conductance in *cax1-1* and *cax3-1* plants, although this was less significant than in *cax1/cax3.* When the length of exposure to HCS was reduced from 18 to 2 hours, we found that the *cax1-1*, but not *cax3-1*, plants had significantly reduced photosynthetic and rosette conductance rates compared with Col-0 (Supplementary Fig. 6). This suggests that the transporters that buffer increases in apoplastic [Ca^2+^] around mesophyll cells in *cax1-1* plants, which include CAX3, are less effective than in wild-type plants containing CAX1, but only in the short term.

### CAX1 and CAX3 interact as homo- and heterodimers *in planta* and facilitate Ca^2+^ transport when co-expressed in yeast

**Fig. 7.**
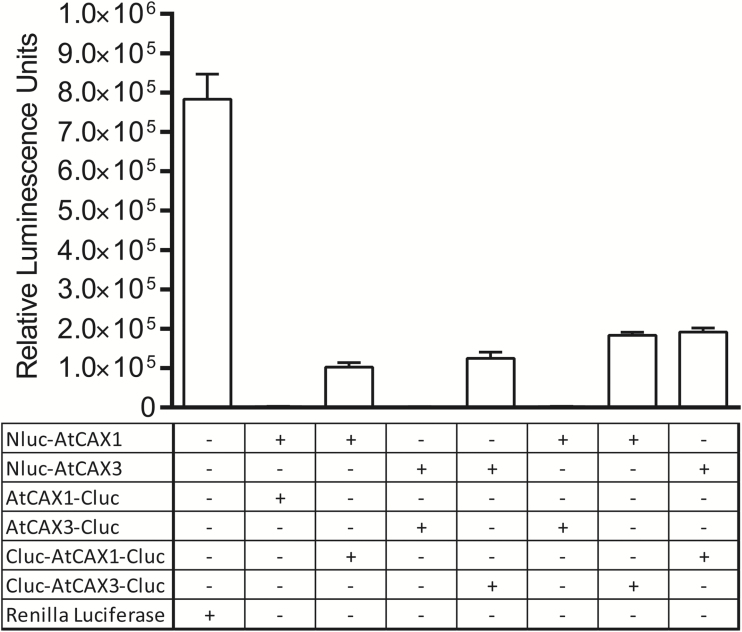
Split luciferase protein–protein interaction assay to determine CAX1 and CAX3 interactions in mesophyll protoplasts (Fujikawa and Kato, 2007). Full-length *CAX1* and *CAX3* lacking a stop codon were recombined into split luciferase vectors with either the N-terminal (Nluc) or C-terminal (Cluc) half of luciferase fused to the N- or C-terminus of CAX1 or CAX3. Full-length Renilla luciferase was used as a positive control.

Despite previous reports identifying the capacity of CAX1 and CAX3 proteins to interact in yeast and in plants under the control of ubiquitous promoters (Zhao *et al.*, 2009*a*, 2009*b*), the nature of their interactions remains largely unexplored. We sought to address this latter point by expressing full-length *CAX1* and *CAX3* using a split luciferase reporter construct in mesophyll protoplasts (Fig. 7). We found that both CAX1 and CAX3 could homodimerize and heterodimerize, and these interactions were abolished by co-transfecting with an artificial miRNA designed against one of the genes (see Supplementary Fig. 7). Furthermore, by switching the split luciferase between the N- and C-terminus, we found that the interaction was mediated by the N-termini of both proteins (head-to-head fashion) (Fig. 7; Supplementary Fig. 7).

**Fig. 8.**
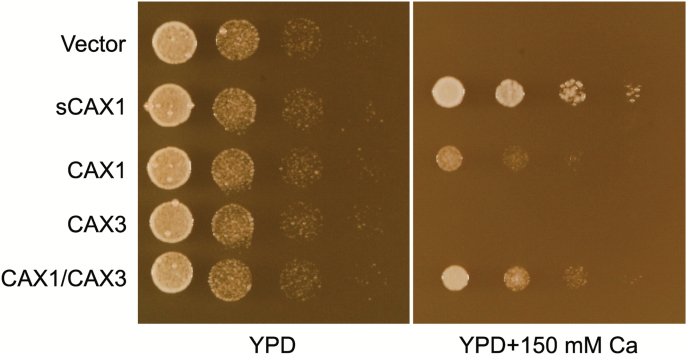
Comparison of phenotypes of yeast cells expressing *sCAX1* and *CAX1-CAX3* transporters, showing suppression of Ca^2+^ sensitivity in yeast mutant cells that are defective in vacuolar Ca^2+^ transport. Suppression assays were performed by spotting dilutions of *CAX*-expressing yeast mutant strains and growing the cells on solid YPD medium and YPD medium containing 150 mM Ca^2+^. This photograph was taken after 3 days of incubation at 30 °C. *sCAX1* is a truncated version of *CAX1* lacking a 36 amino acid N-terminal autoinhibitory domain. *CAX1/CAX3* indicates co-expression of both *CAX1* and *CAX3*.

To gain insights into the functional relevance of the interaction between CAX1 and CAX3, we utilized yeast expression assays. Previously, it has been demonstrated that co-expression of *CAX1* and *CAX3* can suppress yeast vacuolar Ca^2+^ transport defects, whereas expression of either transporter individually fails to do so (Cheng *et al.*, 2005; Manohar *et al.*, 2011*b*). The negative regulatory domains within CAX1 and CAX3 prohibit the functional expression of either transporter when individually expressed in yeast cells (Cheng *et al.*, 2005; Manohar *et al.*, 2011*b*). To avoid potential artifacts arising from the plasmid-dependent CAX overexpression approaches used previously, we modified yeast to integrate both CAX transporters into the genome to ensure stable expression levels. Only strains harboring both constructs conferred Ca^2+^ tolerance to yeast mutants defective in vacuolar Ca^2+^ transport (Fig. 8). Immunoblot analysis demonstrated that both CAX1 and CAX3 proteins accumulated to comparable levels in yeast cells (see Supplementary Fig. 8).

**Fig. 9.**
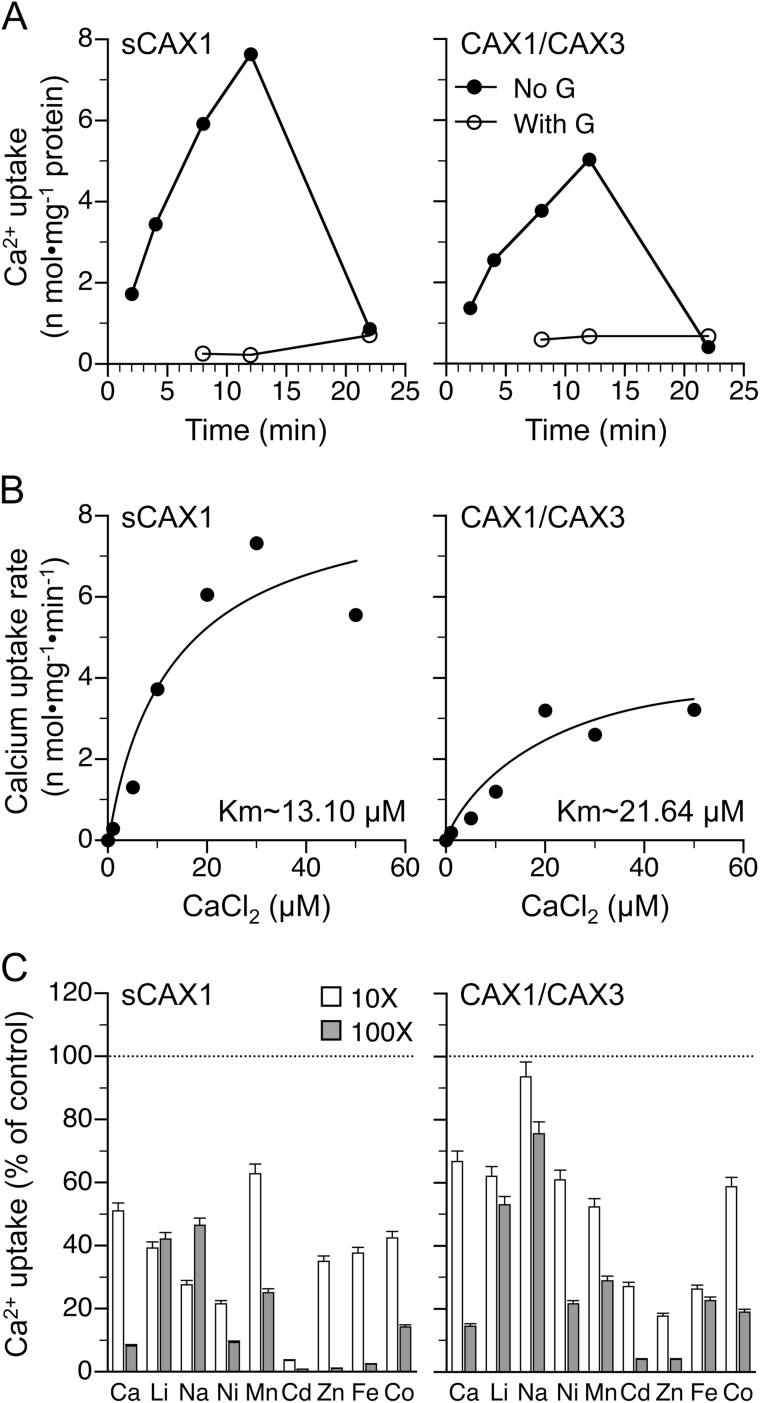
Michaelis–Menten kinetic analysis and comparison of inhibition of Ca^2+^ uptake in the presence of other metals. (A) Time course of ^45^Ca^2+^ uptake into vacuolar vesicles prepared from the yeast strain K667 expressing *sCAX1* or *CAX1/CAX3*. Results are shown in the absence and presence of a protonophore (gramicidin; G) The Ca^2+^ ionophore A23187 (5 µM) was added at 12 min and uptake was measured at 22 min. Data are representative of three independent experiments. (B) Michaelis–Menten kinetic analysis of the initial rate of metal/H^+^ exchange. A preset steady-state pH gradient was generated in vacuolar-enriched vesicles from yeast cells expressing *sCAX1* and *CAX1-CAX3* by activation of the V-ATPase. Initial rates of H^+^-dependent Ca^2+^ uptake were calculated over a range of Ca^2+^ concentrations from 0 to 50 µM. Data are representative of three independent experiments. (C) Inhibition of Ca^2+^ uptake by *sCAX1* or *CAX1-CAX3* into yeast vacuolar-enriched vesicles in the presence of other metal ions. Uncoupler-sensitive (∆pH-dependent) uptake of 10 μM ^45^Ca^2+^ was measured in the absence (control with 100% activity, shown by the dotted line) or presence of 10× or 100× non-radioactive CaCl_2_, LiCl, NaCl, NiSO_4_, MnCl_2_, CdCl_2_, ZnCl_2_, FeCl_3_, or CoCl_2_ after 10 min. Data are averages of at least three replications from two independent membrane preparations, and are presented as mean±SEM.

We measured transport properties by measuring ^45^Ca^2+^ uptake activity in membrane vesicles isolated from yeast cells expressing integrated *CAX1*-*CAX3* and the plasmid-based deregulated *sCAX1*, an artificially truncated form of CAX1 with the autoinhibitory domain removed. In this system, the pH gradient across yeast vacuolar membrane vesicles was generated by activation of the vacuolar H^+^-ATPase. The vesicles of *sCAX1*- and *CAX1*-*CAX3*-expressing cells took up ^45^Ca^2+^ from the medium in a pH- and time-dependent manner for up to 12 min (Fig. 9A). The accumulated ^45^Ca^2+^ was released after the addition of the Ca^2+^ ionophore A23187. The addition of gramicidin, a protonophore that dissipates the pH gradient, eliminated membrane vesicle Ca^2+^ uptake activity. Membrane vesicles of yeast cells expressing an empty vector had negligible activity (data not shown). Interestingly, *CAX1-CAX3*-expressing yeast cells demonstrated transport activity that differed from that of the deregulated *sCAX1*-expressing cells (Fig. 9A). Moreover, Michaelis–Menten kinetic analysis of the data showed that *CAX1*-*CAX3*-expressing cells displayed a *K*_m_ of 21.64 µM for Ca^2+^, while *sCAX1*-expressing cells demonstrated a *K*_m_ of 13.10 µM (Fig. 9B).

To analyze and compare the substrate specificity of the putative CAX1-CAX3 transporters, competition experiments were performed. This approach allowed us to determine the effect of co-expressing CAX proteins in terms of cation selectivity in comparison to the deregulated sCAX1. Initially, we measured Ca^2+^ uptake in *sCAX1*- and *CAX1*-*CAX3*-expressing cells. The pH-dependent 10 µM ^45^Ca^2+^ uptake into yeast microsomal vesicles isolated from strains expressing empty vector (data not shown), *sCAX1*, and *CAX1*-*CAX3* (Fig. 9C) was measured at a single 10 min time point. Ca^2+^ uptake determined in the absence of excess non-radioactive metal (control) was compared with Ca^2+^ uptake determined in the presence of two concentrations (10× and 100× excess) of various non-radioactive metals (Zhao *et al.*, 2008). Inhibition of Ca^2+^ uptake by non-radioactive Ca^2+^ was used as an internal control; as expected, excess Ca^2+^ inhibited Ca^2+^ uptake in both *sCAX1*- and *CAX1-CAX3*-expressing yeast. Non-radioactive Ca^2+^, particularly the 10× concentration, did not completely inhibit Ca^2+^ uptake, further highlighting the low Ca^2+^ affinity of the transporters. Ca^2+^ uptake by *sCAX1*-expressing cells was strongly inhibited by a 10× concentration of Cd^2+^, whereas *CAX1*-*CAX3*-mediated Ca^2+^ transport was only moderately inhibited. Interestingly, microsomes from *CAX1*-*CAX3*-expressing cells, compared with *sCAX1*-expressing cells, displayed less Ca^2+^ uptake inhibition by Li^+^ and Na^+^. These data demonstrate that the CAX1-CAX3 complex has altered Ca^2+^ affinity and transport capacity compared with the deregulated sCAX1. These observations imply that the Ca^2+^ dynamics may be different in plant cells containing the CAX1-CAX3 complex compared with cells containing only CAX1 or CAX3.

## Discussion

We found that both *CAX1* and *CAX3* are expressed in stomatal guard cells, unlike most other leaf tissues, under standard conditions. This corroborates previous studies that have detected *CAX1* and *CAX3* RNA in isolated guard cell protoplast preparations (Leonhardt *et al.*, 2004; Yang *et al.*, 2008). These studies also found *CAX3* to be expressed in the mesophyll. However, when we extracted RNA from non-protoplasted leaf tissue, using LMD or SiCSA, we could not detect *CAX3* transcript in mesophyll cells (Fig. 1A). Only when protoplast incubation was extended to 24 h could *CAX3* expression be detected at moderate levels in the mesophyll (Fig. 1B). The cell wall damage that occurs during protoplasting has been suggested to mimic physiological perturbations that occur in the cell wall in response to pathogen infections (Ecker and Davis, 1987). Our finding that flg22, a bacterial elicitor peptide, stimulates *CAX3* expression in the mesophyll cells supports the conclusion that *CAX3* expression is induced by wounding and pathogens, but is otherwise normally absent from the mesophyll (Table 1; Fig. 3). As both *CAX1* and *CAX3* are present in mesophyll cells in response to bacterial elicitors, and a functional CAX1-CAX3 complex can form, it is likely that the transport properties of this complex could participate in the physiological response to pathogen attack.

It is interesting to note that *cax1/cax3* plants, which have an altered capacity for Ca^2+^ secretion into mesophyll cells, had an increased apoplastic Ca^2+^ concentration, and an altered transcript profile enriched in pathogen-responsive genes (see Supplementary Table S3). For instance, *PR1* and *PR2*, in addition to many cell-wall-related genes, were upregulated in the *cax1/cax3* rosette; expression could be reduced to wild-type levels by transferring plants to a low Ca^2+^ condition (Fig. 6A; Supplementary Fig. S9) (Conn *et al.*, 2011*b*). Altered Ca^2+^ compartmentation into the mesophyll and apoplast may also mimic some plant responses to pathogen infection. For example, heterologous expression of Arabidopsis *sCAX1* in tomato results in upregulated expression of two pathogen-related proteins, *PR* P2 precursor and *PR* leaf protein 4-like, homologs of *PR1* and *PR4* from *A. thaliana* (~8- and ~23-fold, respectively) (De Freitas *et al.*, 2011). Similarly, four *PR* genes were induced in *cax1/cax3* plants, including *PR1* (17-fold) and *PR5* (11-fold) (Conn *et al.*, 2011*b*). The increased membrane leakage and blossom end rot symptoms in tomato fruits were considered to be due to the impact of enhanced vacuolar Ca^2+^ storage on Ca^2+^-signaling-related proteins and disturbed apoplastic [Ca^2+^], as well as cell wall modification (De Freitas *et al.*, 2011). The upregulation of *PR* genes in s*CAX1*-expressing tomato and Arabidopsis *cax1/cax3* lines suggests that a modification in CAX-mediated Ca^2+^ transport in cells may also occur during pathogen responses of plants (Hocking *et al.*, 2016). Variation in the intracellular Ca^2+^ concentration in a plant cell is known to be a critical step for early defense signaling pathways (Lecourieux *et al.*, 2006).

The observation that *CAX1* and *CAX3* are expressed in guard cells (Fig. 4) suggests a role for the CAX1-CAX3 complex in stomatal function. A smaller mean stomatal aperture was found in epidermal strips of *cax1-1*, *cax3-1*, and *cax1/cax3* plants relative to Col-0 (Fig. 5A). These findings are consistent with a previous study Cho *et al.* (2012), which found significantly reduced steady-state stomatal apertures in epidermal strips in *cax1-1*, *cax3-1*, and *cax1/cax3* plants compared with wild-type. These mutants were also non-responsive to increased Ca^2+^ concentrations, indicating that each isoform is required in guard cells for a normal response to apoplastic Ca^2+^ (Fig. 5B). We propose that the CAX1-CAX3 complex may function in modulating apoplastic Ca^2+^ signaling and is required for maintenance of normal stomatal aperture in response to changes in external Ca^2+^ concentration; this might occur through the sensing of external Ca^2+^ signals through the CAS apoplastic sensor pathway or due to misregulation of cytosolic free Ca^2+^ affecting intracellular signaling in the guard cell (Wang *et al.*, 2014).

Interestingly, however, we found no statistical difference in gas exchange rate in the single *cax1-1* or *cax3-1* mutants under most conditions (see Supplementary Figs S5 and S6). Previously, we have demonstrated that mesophyllic CAX1 controls leaf apoplastic [Ca^2+^] and that CAX3 could compensate for loss of CAX1 in *cax1-1* lines (Cheng *et al.*, 2005; Conn *et al.*, 2011*b*). Here, we demonstrate that *CAX3* expression was induced in the mesophyll cells of *cax1-1* plants, substituting for CAX1 (Fig. 1). Under standard Ca^2+^ conditions, ectopic expression of *CAX3* in the mesophyll in *cax1-1* plants prevents excessive Ca^2+^ accumulation in the apoplast and allows the maintenance of growth rate and gas exchange in *cax1-1* plants (Supplementary Figs S3–S5) (Cheng *et al.*, 2003). However, after a 2 h pulse of high Ca^2+^ to the roots of *cax1-1* plants, the gas exchange rates were reduced compared with wild-type or *cax3-1* plants (Supplementary Fig. S6); this indicates that CAX3 cannot fully complement *cax1-1*. This reduction in gas exchange was not observed in *cax1-1* plants when high Ca^2+^ was supplied over 18 h or 7 days, suggesting that the plants can adapt to cope with this Ca^2+^ load over a longer time period.

In this study, we demonstrate that integrated expression of both *CAX1* and *CAX3* can catalyze vacuolar Ca^2+^ uptake and rescue the Ca^2+^-hypersensitive phenotype of yeast strains defective in Ca^2+^ transport (Fig. 8A). Further analysis on yeast vesicles showed that the heteromeric CAX1-CAX3 complex has similar Ca^2+^ transport properties to deregulated CAXs (Fig. 9; Supplementary Fig. S6) (Cheng *et al.*, 2005; Manohar *et al.*, 2011*b*). These yeast assays support the hypothesis that CAX3 may act as an activator of the negatively regulated CAX1 in specific plant tissues (Conn *et al.*, 2011*b*; Cho *et al.*, 2012). The transport affinity of the CAX1-CAX3 complex in yeast assays was different from that of the deregulated transporters and this finding suggests that coupling between CAX transporters could be a mechanism for increasing the range of transporter functions.

## Conclusions

Evidence from non-plant studies is beginning to provide confirmation that CAX proteins are able to modulate Ca^2+^ signals (Guttery *et al.*, 2013; Melchionda *et al.*, 2016). These studies have the advantage that the CAX proteins are encoded by single genes, and therefore genetic dissection of CAX Ca^2+^ signaling is not hampered by genetic redundancy. Our study highlights that the multigene CAX families found throughout the plant kingdom may allow the formation of complex functional heteromeric complexes. In yeast-based assays, CAX1-CAX3 displayed transport properties that could not be recreated by high-level expression of either native transporter individually. We also investigated the significance of these interactions for a variety of plant physiological responses. We found that the CAX1-CAX3 complex can occur in leaf mesophyll in response to pathogen attack. Additionally, CAX3 and the CAX1-CAX3 complex may be important in guard cells for maintenance of normal calcium responses and signaling pathways. Further work is required to determine the full extent of the signaling pathways in which CAX1-CAX3 may play a role.

## Supplementary data

Supplementary data are available at *JXB* online.

Fig. S1. Profiling *CAX1* and *CAX3* promoter activity in mesophyll protoplasts.

Fig. S2. qPCR on whole-leaf RNA.

Fig. S3. Mesophyll vacuolar and leaf apoplastic Ca^2+^ concentrations.

Fig. S4. Physiological parameters affected by changes in [Ca^2+^]_apo_.

Fig. S5. Gas exchange rates for Col-0, *cax1-1*, *cax3-1*, and *cax1/cax3* with and without 18 h Ca^2+^ treatment.

Fig. S6. Gas exchange rates for Col-0, *cax1-1*, *cax3-1*, and *cax1/cax3* after 2 h of Ca^2+^ treatment.

Fig. S7. Split luciferase protein–protein assay determining whether CAX1 and CAX3 interact.

Fig. S8. Western blots showing relative levels of CAX1 and CAX3.

Fig. S9. Expression of *PR1* and *PR2* in 6-week-old Col-0 and *cax1/cax3* shoots.

Table S1. Contents of media used for growth studies: BNS, HCS, and SLCS.

Table S2. PCR primers used in this study.

Table S3. Pathogen-related genes that are differentially expressed in *cax1/cax3* plants compared with Col-0.

## Supplementary Material

Supplementary_Tables_S1-S3_Figures_S1-S9

## References

[CIT0001] Athman A, Tanz SK, Conn VM, Jordans C, Mayo GM, Ng WW, Burton RA, Conn SJ, Gilliham M 2014 Protocol: a fast and simple in situ PCR method for localising gene expression in plant tissue. Plant Methods 10, 29. 25250056 10.1186/1746-4811-10-29 PMC4171716

[CIT0002] Austin RS, Hiu S, Waese J 2016 New BAR tools for mining expression data and exploring Cis-elements in *Arabidopsis thaliana*. The Plant Journal 88, 490–504. 27401965 10.1111/tpj.13261

[CIT0003] Catalá R, Santos E, Alonso JM, Ecker JR, Martinez-Zapater JM, Salinas J 2003 Mutations in the Ca^2+^/H^+^ transporter CAX1 increase CBF/DREB1 expression and the cold-acclimation response in *Arabidopsis*. The Plant Cell 15, 2940–2951. 14630965 10.1105/tpc.015248 PMC282833

[CIT0004] Cheng NH, Pittman JK, Barkla BJ, Shigaki T, Hirschi KD 2003 The *Arabidopsis* cax1 mutant exhibits impaired ion homeostasis, development, and hormonal responses and reveals interplay among vacuolar transporters. The Plant Cell 15, 347–364. 12566577 10.1105/tpc.007385 PMC141206

[CIT0005] Cheng NH, Pittman JK, Shigaki T, Hirschi KD 2002 Characterization of CAX4, an *Arabidopsis* H^+^/cation antiporter. Plant Physiology 128, 1245–1254. 11950973 10.1104/pp.010857 PMC154252

[CIT0006] Cheng NH, Pittman JK, Shigaki T, Lachmansingh J, LeClere S, Lahner B, Salt DE, Hirschi KD 2005 Functional association of *Arabidopsis* CAX1 and CAX3 is required for normal growth and ion homeostasis. Plant Physiology 138, 2048–2060. 16055687 10.1104/pp.105.061218 PMC1183394

[CIT0007] Cho D, Villiers F, Kroniewicz L, Lee S, Seo YJ, Hirschi KD, Leonhardt N, Kwak JM 2012 Vacuolar CAX1 and CAX3 influence auxin transport in guard cells via regulation of apoplastic pH. Plant Physiology 160, 1293–1302. 22932758 10.1104/pp.112.201442 PMC3490596

[CIT0008] Conn S, Gilliham M 2010 Comparative physiology of elemental distributions in plants. Annals of Botany 105, 1081–1102. 20410048 10.1093/aob/mcq027 PMC2887064

[CIT0009] Conn SJ, Conn V, Tyerman SD, Kaiser BN, Leigh RA, Gilliham M 2011a Magnesium transporters, MGT2/MRS2-1 and MGT3/MRS2-5, are important for magnesium partitioning within *Arabidopsis thaliana* mesophyll vacuoles. New Phytologist 190, 583–594. 21261624 10.1111/j.1469-8137.2010.03619.x

[CIT0010] Conn SJ, Gilliham M, Athman A 2011b Cell-specific vacuolar calcium storage mediated by CAX1 regulates apoplastic calcium concentration, gas exchange, and plant productivity in *Arabidopsis*. The Plant Cell 23, 240–257. 21258004 10.1105/tpc.109.072769 PMC3051233

[CIT0011] Conn SJ, Hocking B, Dayod M 2013 Protocol: optimising hydroponic growth systems for nutritional and physiological analysis of *Arabidopsis thaliana* and other plants. Plant Methods 9, 4. 23379342 10.1186/1746-4811-9-4 PMC3610267

[CIT0012] Connorton JM, Webster RE, Cheng N, Pittman JK 2012 Knockout of multiple *Arabidopsis* cation/H^+^ exchangers suggests isoform-specific roles in metal stress response, germination and seed mineral nutrition. PLoS One 7, e47455. 23071810 10.1371/journal.pone.0047455 PMC3470555

[CIT0013] Cunningham KW, Fink GR 1996 Calcineurin inhibits VCX1-dependent H^+^/Ca^2+^ exchange and induces Ca2^+^ ATPases in *Saccharomyces cerevisiae*. Molecular and Cellular Biology 16, 2226–2237. 8628289 10.1128/mcb.16.5.2226 PMC231210

[CIT0014] De Freitas ST, Padda M, Wu Q, Park S, Mitcham EJ 2011 Dynamic alternations in cellular and molecular components during blossom-end rot development in tomatoes expressing sCAX1, a constitutively active Ca^2+^/H^+^ antiporter from *Arabidopsis*. Plant Physiology 156, 844–855. 21464475 10.1104/pp.111.175208 PMC3177280

[CIT0015] Dodd AN, Kudla J, Sanders D 2010 The language of calcium signaling. Annual Review of Plant Biology 61, 593–620. 10.1146/annurev-arplant-070109-104628 20192754

[CIT0016] Ecker JR, Davis RW 1987 Plant defense genes are regulated by ethylene. Proceedings of the National Academy of Sciences of the United States of America 84, 5202–5206. 16593860 10.1073/pnas.84.15.5202 PMC298822

[CIT0017] Fujikawa Y, Kato N 2007 Split luciferase complementation assay to study protein–protein interactions in *Arabidopsis* protoplasts. The Plant Journal 52, 185–195. 17662028 10.1111/j.1365-313X.2007.03214.x

[CIT0018] Gilliham M, Dayod M, Hocking BJ, Xu B, Conn SJ, Kaiser BN, Leigh RA, Tyerman SD 2011 Calcium delivery and storage in plant leaves: exploring the link with water flow. Journal of Experimental Botany 62, 2233–2250. 21511913 10.1093/jxb/err111

[CIT0019] Guttery DS, Pittman JK, Frénal K 2013 The *Plasmodium berghei* Ca^2+^/H^+^ exchanger, PbCAX, is essential for tolerance to environmental Ca^2+^ during sexual development. PLoS Pathogens 9, e1003191. 23468629 10.1371/journal.ppat.1003191 PMC3585132

[CIT0020] Hetherington AM, Brownlee C 2004 The generation of Ca^2+^ signals in plants. Annual Review of Plant Biology 55, 401–427. 10.1146/annurev.arplant.55.031903.141624 15377226

[CIT0021] Hirschi KD, Zhen R-G, Cunningham KW, Rea PA, Fink GR 1996 CAX1, an H^+^/Ca^2+^ antiporter from *Arabidopsis*. Proceedings of the National Academy of Sciences of the United States of America 93, 8782–8786. 8710949 10.1073/pnas.93.16.8782 PMC38751

[CIT0022] Hocking B, Tyerman SD, Burton RA, Gilliham M 2016 Fruit calcium: transport and physiology. Frontiers in Plant Science 7, 569. 27200042 10.3389/fpls.2016.00569 PMC4850500

[CIT0023] Lecourieux D, Ranjeva R, Pugin A 2006 Calcium in plant defence-signalling pathways. New Phytologist 171, 249–269. 16866934 10.1111/j.1469-8137.2006.01777.x

[CIT0024] Leonhardt N, Kwak JM, Robert N, Waner D, Leonhardt G, Schroeder JI 2004 Microarray expression analyses of *Arabidopsis* guard cells and isolation of a recessive abscisic acid hypersensitive protein phosphatase 2C mutant. The Plant Cell 16, 596–615. 14973164 10.1105/tpc.019000 PMC385275

[CIT0025] Li JF, Park E, von Arnim AG, Nebenführ A 2009 The FAST technique: a simplified *Agrobacterium*-based transformation method for transient gene expression analysis in seedlings of Arabidopsis and other plant species. Plant Methods 5, 6. 19457242 10.1186/1746-4811-5-6 PMC2693113

[CIT0026] Manohar M, Shigaki T, Hirschi KD 2011a Plant cation/H^+^ exchangers (CAXs): biological functions and genetic manipulations. Plant Biology 13, 561–569. 21668596 10.1111/j.1438-8677.2011.00466.x

[CIT0027] Manohar M, Shigaki T, Mei H, Park S, Marshall J, Aguilar J, Hirschi KD 2011b Characterization of *Arabidopsis* Ca^2+^/H^+^ exchanger CAX3. Biochemistry 50, 6189–6195. 21657244 10.1021/bi2003839

[CIT0028] Melchionda M, Pittman JK, Mayor R, Patel S 2016 Ca^2+^/H^+^ exchange by acidic organelles regulates cell migration in vivo. Journal of Cell Biology 212, 803–813. 27002171 10.1083/jcb.201510019 PMC4810305

[CIT0029] Nakagawa T, Suzuki T, Murata S 2007 Improved Gateway binary vectors: high-performance vectors for creation of fusion constructs in transgenic analysis of plants. Bioscience, Biotechnology, and Biochemistry 71, 2095–2100. 10.1271/bbb.70216 17690442

[CIT0030] Pittman J, Hirschi K 2016 CAX-ing a wide net: cation/H^+^ transporters in metal remediation and abiotic stress signalling. Plant Biology 18, 741–749. 27061644 10.1111/plb.12460 PMC4982074

[CIT0031] Pittman JK, Hirschi KD 2003 Don’t shoot the (second) messenger: endomembrane transporters and binding proteins modulate cytosolic Ca^2+^ levels. Current Opinion in Plant Biology 6, 257–262. 12753975 10.1016/s1369-5266(03)00036-0

[CIT0032] Pittman JK, Shigaki T, Hirschi KD 2005 Evidence of differential pH regulation of the *Arabidopsis* vacuolar Ca^2+^/H^+^ antiporters CAX1 and CAX2. FEBS Letters 579, 2648–2656. 15862304 10.1016/j.febslet.2005.03.085

[CIT0033] Plett D, Safwat G, Gilliham M, Skrumsager Møller I, Roy S, Shirley N, Jacobs A, Johnson A, Tester M 2010 Improved salinity tolerance of rice through cell type-specific expression of AtHKT1;1. PLoS One 5, e12571. 20838445 10.1371/journal.pone.0012571 PMC2933239

[CIT0034] Pottosin II, Schönknecht G 2007 Vacuolar calcium channels. Journal of Experimental Botany 58, 1559–1569. 17355948 10.1093/jxb/erm035

[CIT0035] Schmittgen TD, Livak KJ 2008 Analyzing real-time PCR data by the comparative *C*_T_ method. Nature Protocols 3, 1101–1108. 18546601 10.1038/nprot.2008.73

[CIT0036] Schwab R, Ossowski S, Riester M, Warthmann N, Weigel D 2006 Highly specific gene silencing by artificial microRNAs in *Arabidopsis*. The Plant Cell 18, 1121–1133. 16531494 10.1105/tpc.105.039834 PMC1456875

[CIT0037] Sherman F, Fink GR, Hicks JB 1986 Laboratory course manual for methods in yeast genetics. Cold Spring Harbor: Cold Spring Harbor Laboratory.

[CIT0038] Shigaki T, Cheng NH, Pittman JK, Hirschi K 2001 Structural determinants of Ca^2+^ transport in the *Arabidopsis* H^+^/Ca^2+^ antiporter CAX1. Journal of Biological Chemistry 276, 43152–43159. 11562366 10.1074/jbc.M106637200

[CIT0039] Shigaki T, Hirschi K 2000 Characterization of *CAX*-like genes in plants: implications for functional diversity. Gene 257, 291–298. 11080595 10.1016/s0378-1119(00)00390-5

[CIT0040] Wang WH, Chen J, Liu TW, Chen J, Han AD, Simon M, Dong XJ, He JX, Zheng HL 2014 Regulation of the calcium-sensing receptor in both stomatal movement and photosynthetic electron transport is crucial for water use efficiency and drought tolerance in *Arabidopsis*. Journal of Experimental Botany 65, 223–234. 24187420 10.1093/jxb/ert362 PMC3883291

[CIT0041] White PJ, Broadley MR 2003 Calcium in plants. Annals of Botany 92, 487–511. 12933363 10.1093/aob/mcg164 PMC4243668

[CIT0042] Yang Y, Costa A, Leonhardt N, Siegel RS, Schroeder JI 2008 Isolation of a strong *Arabidopsis* guard cell promoter and its potential as a research tool. Plant Methods 4, 6. 18284694 10.1186/1746-4811-4-6 PMC2323621

[CIT0043] Yoo SD, Cho YH, Sheen J 2007 *Arabidopsis* mesophyll protoplasts: a versatile cell system for transient gene expression analysis. Nature Protocols 2, 1565–1572. 17585298 10.1038/nprot.2007.199

[CIT0044] Zhao J, Barkla BJ, Marshall J, Pittman JK, Hirschi KD 2008 The *Arabidopsis cax3* mutants display altered salt tolerance, pH sensitivity and reduced plasma membrane H^+^-ATPase activity. Planta 227, 659–669. 17968588 10.1007/s00425-007-0648-2

[CIT0045] Zhao J, Connorton JM, Guo Y, Li X, Shigaki T, Hirschi KD, Pittman JK 2009a Functional studies of split *Arabidopsis* Ca^2+^/H^+^ exchangers. Journal of Biological Chemistry 284, 34075–34083. 19819871 10.1074/jbc.M109.070235 PMC2797178

[CIT0046] Zhao J, Shigaki T, Mei H, Guo YQ, Cheng NH, Hirschi KD 2009b Interaction between *Arabidopsis* Ca^2+^/H^+^ exchangers CAX1 and CAX3. Journal of Biological Chemistry 284, 4605–4615. 19098009 10.1074/jbc.M804462200

