# Supplementary material for: Heterodimerization of Arabidopsis calcium/proton exchangers contributes to regulation of guard cell dynamics and plant defense responses
Source: J Exp Bot. 2017 Jun 22;68(15):4171–83. doi: 10.1093/jxb/erx209 (PMC5853972; doi:10.1093/jxb/erx209)
Supplement: Supplementary_Tables_S1-S3_Figures_S1-S9 [file erx209_suppl_supplementary_tables_s1-s3_figures_s1-s9.pdf]

Heterodimerisation of Arabidopsis calcium/proton exchangers contributes to regulation of guard cell dynamics and plant defense responses

Bradleigh Hocking, Simon J. Conn, Murli Manohar, Bo Xu, Asmini Athman, Matthew A Stancombe, Alex R. Webb, Kendal D. Hirschi, Matthew Gilliham

Supplemental Tables and Figures

Supplementary Table 1. Contents of media used for growth studies: BNS, HCS and SLCS

Nutrients	Concentration		
	BNS	HCS	SLCS
Macronutrients (mM)			
K	5.6	5.9	5.6
Ca	2.1	10.85	0.3
Mg	2	2.5	2
NH_4	2	2.1	2
Cl	3.71	18.314	3.51
NO_3	9	9.7	9
SO_4	2.0105	3.0107	2.0105
PO_4	0.6	0.7	0.6
Na	1.6012	1.6522	5.0012
Micronutrients (μM)			
Fe	10	10	10
Mn	5	5	5
Zn	10	10	10
Cu	0.5	0.5	0.5
Mo	0.1	0.1	0.1

Supplementary Table 2. Primers used in this study

Gene	Primer Name	Primer Sequence (5'-3')	Techniques
CAX1 (At2g38170)	CAX1_qF	CATCATCGTGGCGTGGATT	qPCR analysis
	CAX1_qR	GCATTTTGTTTCTGGGGAAGT	
CAX3 (At3g51860)	CAX3_qF	ACTGGTTCTATTGTTATGCTATGTCA	
	CAX3_qR	CAAGCTCCCTCCCTCATTC	
Actin2 (At3g18780)	Actin2_qF	TGAGCAAAGAAATCACAGCACT	
	Actin2_qR	CCTGGACCTGCCTCATCATAC	
EF1-α (At1g07940)	EF1-α_qF	GTGAAAGGGAGGCGGTGAA	
	EF1-α_qR	ATCACAGTCAAAGGCACATTACAA	
β-Tubulin5 (At1g20010)	β-Tubulin5_qF	CATTCACGCAGGAGGCAGAG	
	β-Tubulin5_qR	CCACACGGCAGAGTTACACTTG	
PR1 (At2g14610)	PR1_qF	GCTCTTGTAGGTGCTCTTGTTCT	
	PR1_qR	TGCCTCTTAGTTGTTCTGCGT	
PR2 (At3g57260)	PR2_qF	TGAGAATAAGAAGGAACCAACG	
	PR2_qR	AACAACATACTACACGCTGAAAG	
GAPDH-A(At3g26650)	GAPDH-A_qF	TGGTTGATCTCGTTGTGCAGGTCTC	
	GAPDH-A_qR	GTCAGCCAAGTCAACAACTCTCTG	
GC1 (At1g22690)	GC1_qF	CGAATGACGAGAACCAAACT	
	GC1_qR	CACAACAACTTCCACAGGCT	
CAX1 promoter (2kb)	pCAX1_F	ACTGCAGTGCAGTTTACACAC	Promoter cloning
	pCAX1_R	TTCTCTACTGACTCAAAACTTTG	
CAX1 promoter truncation F1 (589 bp)	pCAX1_F1	GAAGGCAAATAACATCAATAAGA	
	pCAX1_R1	TTCTCTACTGACTCAAAACTTTGT	
CAX1 promoter truncation F2(953 bp)	proCAX1_F2	ATTCTCCGGTTTACTGTGTATAA	
	proCAX1_R	TTCTCTACTGACTCAAAACTTTGT	
CAX1 promoter truncation F3(1600 bp)	proCAX1_F3	CTTGATTTATTCATGACATCTTTC	
	proCAX1_R	TTCTCTACTGACTCAAAACTTTGT	
CAX3 promoter (2kb)	pCAX3_F	CAAGTCCCTTTTTATCAGCTCTAG	
	pCAX3_R	TACGTGTTATTGTTTTGGTTTATGT	
CAX3 promoter truncation F1 (538 bp)	proCAX3_F1	ACAAACGGATAGTCGGATACC	
	proCAX3_R	GTTTTAAGACGTTTTGATTCTACTACA	
CAX3 promoter truncation F2 (892 bp)	proCAX3_F2	TACATAAACACATACACTGACCACC	
	proCAX3_R	GTTTTAAGACGTTTTGATTCTACTACA	
CAX3 promoter truncation F3 (1555 bp)	proCAX3_F3	AGCCGCTTATCGCCTTTAT	
	proCAX3_R	GTTTTAAGACGTTTTGATTCTACTACA	
CAX1 promoter for pSIM1 construction	pCAX1_*Xho*I_F	TCGAACTCGAGACTGCAGTGCAGTTTACACAC	
	pCAX1_*Hind*IIIR	TATCAAGCTTTTCTCTACTGACTCAAAACTTTG	
CAX3 promoter for pSIM3 construction	pCAX3_*Xho*I_F	CGTTCTCGAGTTTACAACTACATAAGTTTTGGAG	
	pCAX3_*EcoR*V_R	TCCAGATATCGTTTTAAGACGTTTTGATTCTAC	
CAX1 (At2g38170)	CAX1_CDS_F	ATGGCGGGAATCGTGACAGAG	Gene cloning
	CAX1_CDS_R	CTAACGCAACTCCCAAAGATATGTC	
CAX1 (At2g38170) without stop condon	CAX1_CDS_F	ATGGCGGGAATCGTGACAGAG	
	CAX1_CDS_R-stop	ACGCAACTCCCAAAGATATGTCCTAG	
CAX3 (At3g51860)	CAX3_CDS_F	ATGGGAAGTATCGTGGAGCC	
	CAX3_CDS_R	TTAAGCTGAGAAAACTTCTCCC	
CAX3 (At3g51860) without stop condon	CAX3_CDS_F	ATGGGAAGTATCGTGGAGCC	
	CAX3_CDS_R-stop	AGCTGAGAAAACTTCTCCCAAATTG	
Hemagglutinin tag	HAtag_BglII_F	TCAAGAGATCTATCTACCCATACGATGTTCCAGATTAC	HA tagging construct
	HAtag_BglII_R	TCTAGATAGATCTAGCGTAATCTGGAACATCGTATGG	

Supplementary Table 3. Pathogen related genes that are differential expressed in *cax1/cax3* plants compared to Col-0. Data extracted from microarray (Conn *et al.*, 2011b).

Gene Title	Transcript ID	*cax1/cax3*	adj.P.Val
pathogenesis-related protein 1 (PR-1)	At2g14610	-4.1	>0.001
glycosyl hydrolase family 17 protein	At3g57260	-4.08	>0.001
calmodulin-like protein 41 (CML41)	At3g50770	-3.85	<0.001
disease resistance family protein	At2g32680	-3.68	>0.001
pathogenesis-related protein 5 (PR-5)	At1g75040	-3.53	>0.001
disease resistance family protein	At3g25010	-3.03	>0.001
receptor-like protein kinase, putative	At3g45860	-2.88	>0.001
plastocyanin-like domain-containing protein	At5g20230	-2.86	0.01
calmodulin-binding protein	At5g26920	-2.7	>0.001
receptor-like protein kinase, putative	At4g23310	-2.41	>0.001
pseudogene, similar to NL0D	At4g13900	-2.31	>0.001
peroxidase, putative	At5g64120	-2.21	>0.001
disease resistance protein (TIR class), putative	At2g32140	-2.12	>0.001
trypsin inhibitor, putative	At2g43510	-2.11	0.01
WRKY family transcription factor	At4g23810	-1.85	0.01
disease resistance family protein	At3g23110	-1.84	0.04
syntaxin, putative (SYP122)	At3g52400	-1.81	>0.001
isochorismate synthase 1 (ICS1) / isochorismate mutase	At1g74710	-1.79	0.01
calcium-binding EF hand family protein	At1g76650	-1.78	>0.001
WRKY family transcription factor	At4g31800	-1.75	>0.001
patatin, putative	At2g26560	-1.74	>0.001
cytochrome P450, putative	At5g57220	-1.74	>0.001
disease resistance protein (TIR-NBS-LRR class), putative	At5g41740	-1.7	>0.001
disease resistance protein (CC-NBS-LRR class), putative	At1g33560	-1.68	>0.001
phytoalexin-deficient 4 protein (PAD4)	At3g52430	-1.67	>0.001
disease resistance family protein / LRR family protein	At3g24900	-1.59	>0.001
disease resistance protein (TIR-NBS-LRR class), putative	At1g63750	-1.56	>0.001
harpin-induced family protein / HIN1 family protein 3	At5g06320	-1.51	>0.001
peroxidase 21 (PER21) (P21) (PRXR5)	At2g37130	-1.45	0.01
disease resistance protein (TIR class), putative	At1g57630	-1.44	0.01
auxin-responsive GH3 family protein	At5g13320	-1.43	>0.001
expressed protein	At5g50200	-1.42	>0.001
WRKY family transcription factor	At2g38470	-1.36	0.02
disease resistance protein (CC-NBS-LRR class), putative	At4g33300	-1.32	>0.001
thioredoxin H-type 5 (TRX-H-5) (TOUL)	At1g45145	-1.31	0.01
peroxidase 33 (PER33) (P33) (PRXCA) / neutral peroxidase C (PERC)	At3g49120	-1.31	0.01
disease resistance protein (TIR-NBS class), putative	At1g66090	-1.28	0.02
glutathione S-transferase, putative	At1g02930	-1.27	0.01
beta-fructosidase (BFRUCT1) / beta-fructofuranosidase / cell wall invertase	At3g13790	-1.25	>0.001
protein kinase, putative	At2g39660	-1.22	>0.001
WRKY family transcription factor	At2g25000	-1.13	0.02
plant defensin-fusion protein, putative (PDF2.2)	At2g02100	-1.09	0.01
strictosidine synthase family protein	At1g74020	-1.07	>0.001
myb family transcription factor (MYB51)	At1g18570	-1.06	0.02
disease resistance protein (TIR-NBS class), putative	At4g16990	-1.03	0.01
protein kinase family protein	At4g23190	-1.02	>0.001
disease resistance protein (CC-NBS-LRR class), putative	At5g04720	-1.02	>0.001
disease resistance protein (EDS1)	At3g48090	-1.02	0.01
steroid sulfotransferase, putative	At2g03760	-1.01	>0.001
4-coumarate–CoA ligase 3 / 4-coumaroyl-CoA synthase 3 (4CL3)	At1g65060	1.22	0.01
chalcone synthase / naringenin-chalcone synthase	At5g13930	1.64	0.01
cellulose synthase, catalytic subunit (IRX1)	At4g18780	2.01	>0.001
cellulose synthase, catalytic subunit (IRX5)	At5g44030	2.04	>0.001
disease resistance-responsive family protein / dirigent family protein	At4g23690	2.05	>0.001

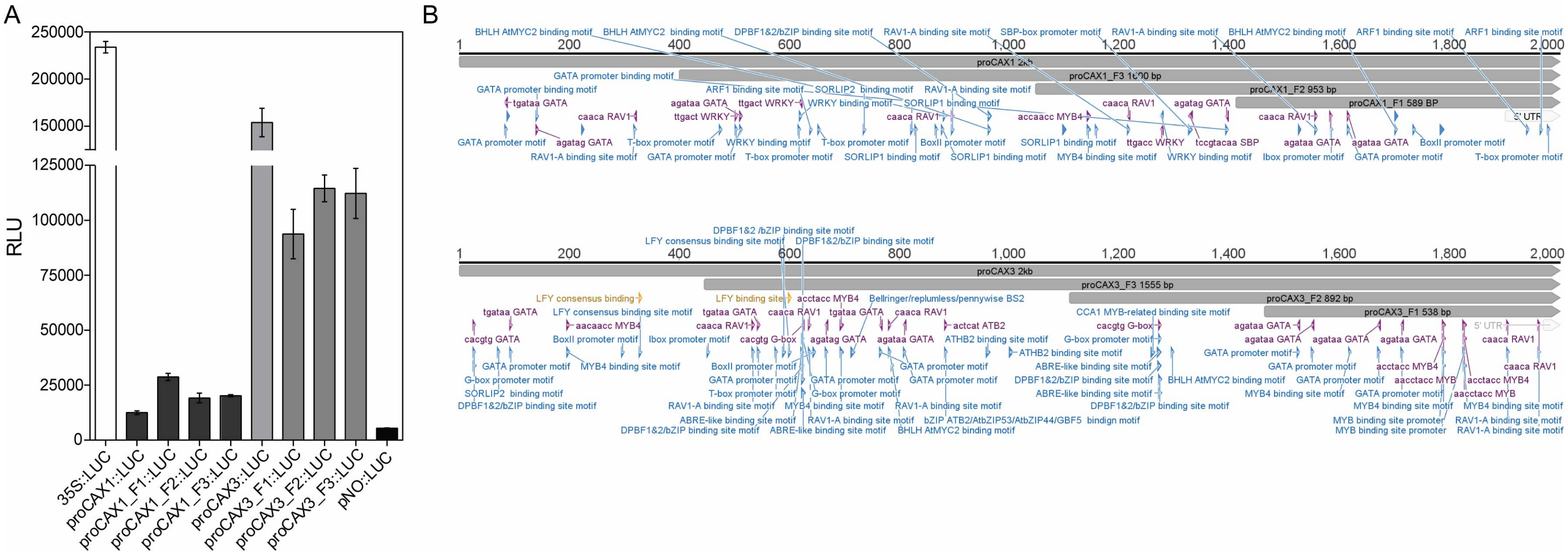

Supplementary Figure 1. Profiling *CAX1* and *CAX3* promoter activity in mesophyll protoplasts. (A) Expression of *CAX* native promoter (full length and fragments) luciferase fusions in Col-0 mesophyll protoplasts. (B) Map full-length *CAX1* and *CAX3* promoters demonstrating truncations and *in silico* analysis of promoter elements.

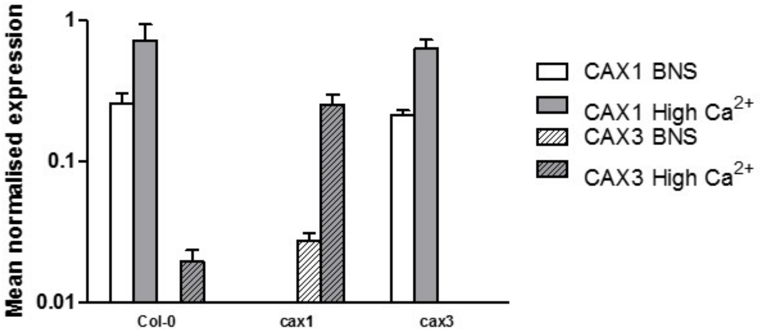

Supplementary Figure 2. qPCR on whole-leaf RNA from 7-week old BNS-grown and HCS-grown Arabidopsis detecting A. *CAX1* and B. *CAX3* expression. Mean normalised expression levels of *CAX1* and *CAX3* in the leaves of BNS- and high Ca^{2+}-treated Col-0 (wild-type), *cax1-1* and *cax3-1* Arabidopsis plants. Calcium concentration in hydroponics as solutions were 2 mM Ca^{2+} (BNS) and 11 mM Ca^{2+} (HCS), with the HCS-treatment for 18 hours. Three semi-quantitative PCR reactions were performed per sample (technical replicates) upon cDNA isolated from three plants per treatment (biological replicates) (n=3), normalization used *Actin2* (At3g18780). Mean ± S.E.M. Primers listed in Supplemental Table 2.

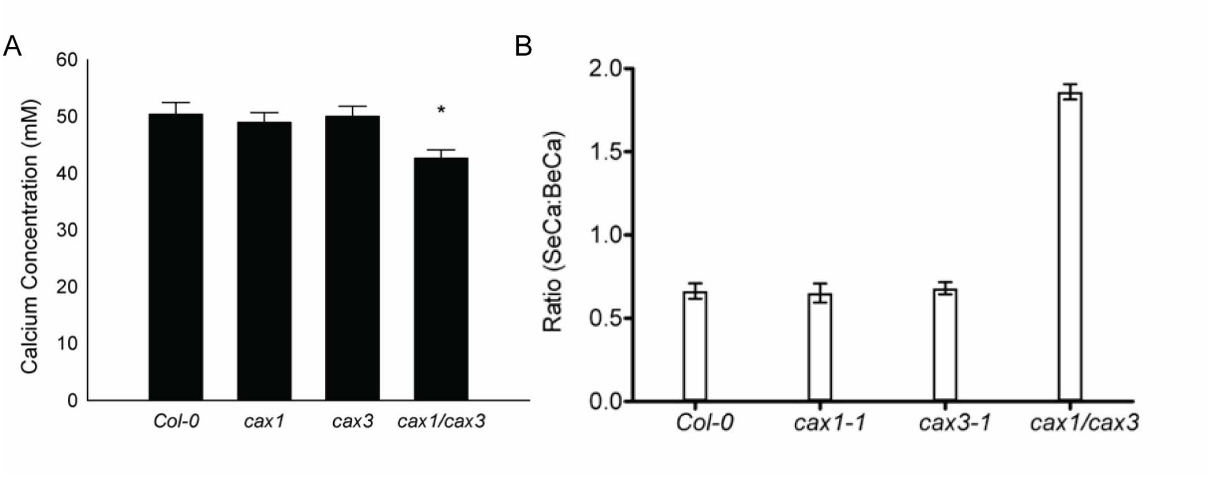

Supplementary Figure 3. (A) Mesophyll vacuolar and (B) leaf apoplastic calcium concentrations of Col-0, *cax1-1, cax3-1* and *cax1/cax3* plants presented as mM or the ratio of 'free' Ca^{2+} to 'bound' Ca^{2+} (SeCa:BeCa), respectively. Measurements performed on six-week old Arabidopsis plants and presented as Mean ratio ± S.E.M. (n = 6 plants), from triplicate experiments. Vacuolar Ca^{2+} captured using SiSCA, and apoplastic Ca^{2+} captured using centrifugation (Conn *et al.*, 2011b). Apoplastic ratios calculated between leaves of the same plant, permitting statistical analysis on biological replicates by 1-way ANOVA with Tukey's posthoc test ($P < 0.01$).

	6 weeks in standard Ca growth solution		5 weeks in standard growth solution + 1 week in reduced Ca growth solution	
	wildtype	*cax1/cax3*	wildtype	*cax1/cax3*
	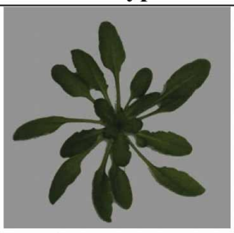	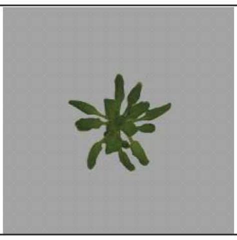	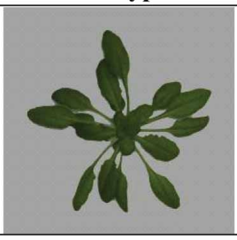	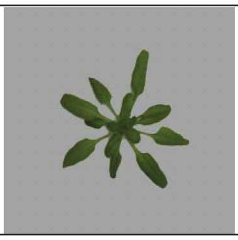
**(1) $[Ca^{2+}]_{apo}$**		+++	-	-
**(2) Growth rate**		---	-	-
**(3) Stomatal Conductance**		--	=	=
**(4) CO_2 assimilation**		--	=	=
**(5) Cell wall extensibility**		-	=	=
**(6) Low DME HGA**		+	=	=

Supplementary Figure 4. Physiological parameters affected by changes in $[Ca^{2+}]_{apo}$. After 6 weeks growth in BNS (Ca^{2+} concentration = 2 mM) or 5 weeks in standard growth solution plus 1 week in standard Ca^{2+} growth solution (LCS, Ca^{2+} concentration 50 µM). Parameters (1)-(6) were measured as detailed in Conn *et al.*, (2011a). +/- Qualitative indication of quantitative data showing significant differences relative to Col-0 grown in standard $[Ca^{2+}]_{ext}$ ($P < 0.05$, no significant difference indicated by =).

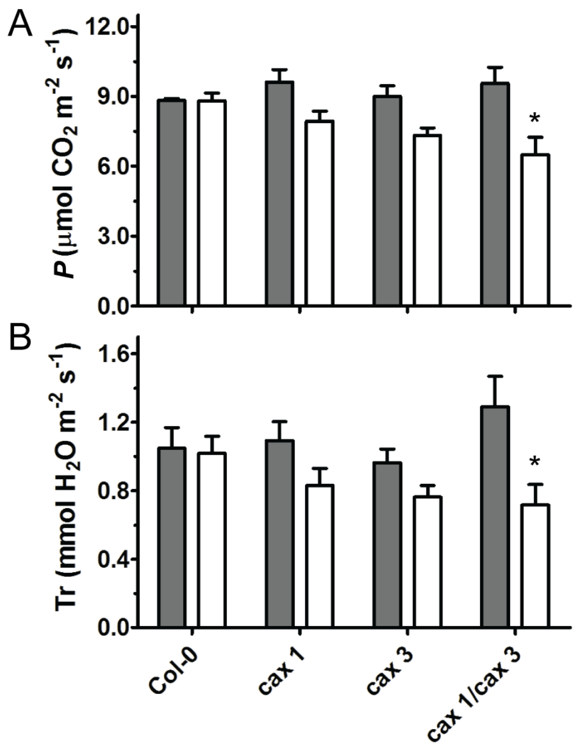

Supplemental Figure 5. Gas exchange rates for Col-0, *cax1-1*, *cax3-1* and *cax1/cax3* with and without 18 hour Ca treatment. Rates are comparable in all lines grown in SLCS (closed bars). 18 hours treatment in HCS (open bars) induced significant ($P < 0.05$) reduction in both *P* and Tr only in double mutants. Treatments were imposed on 6-week old plants for each line. Data shown are Means ± S.E.M of four biological replicates. Asterisk indicates significant difference from Col-0 control, 2-way ANOVA, with Tukey's multicomparisons test ($P < 0.01$).

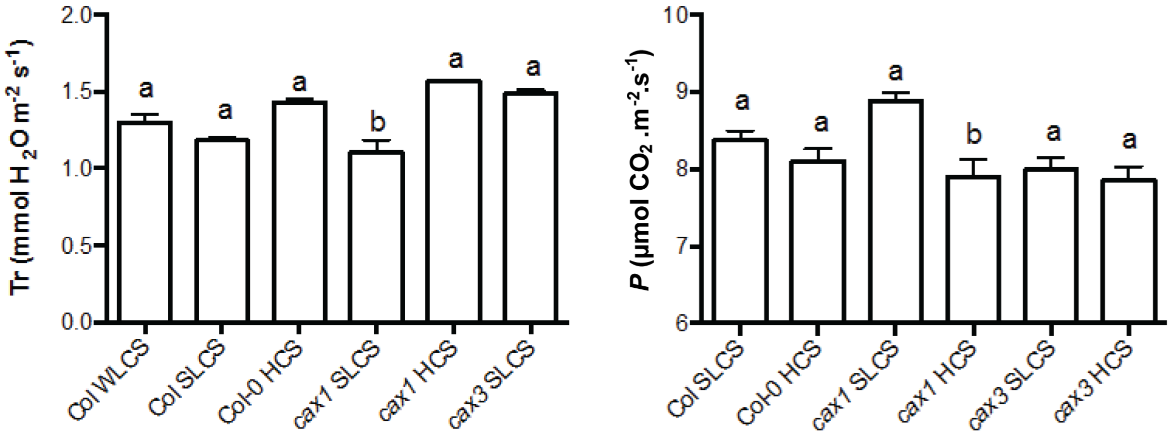

Supplemental Figure 6. Gas exchange rates for Col-0, *cax1-1*, *cax3-1* and *cax1/cax3* after 2 h of Ca treatment. a, b indicates groups with insignificant difference, determined by 1-way ANOVA with Tukey's posthoc test ($P < 0.05$).

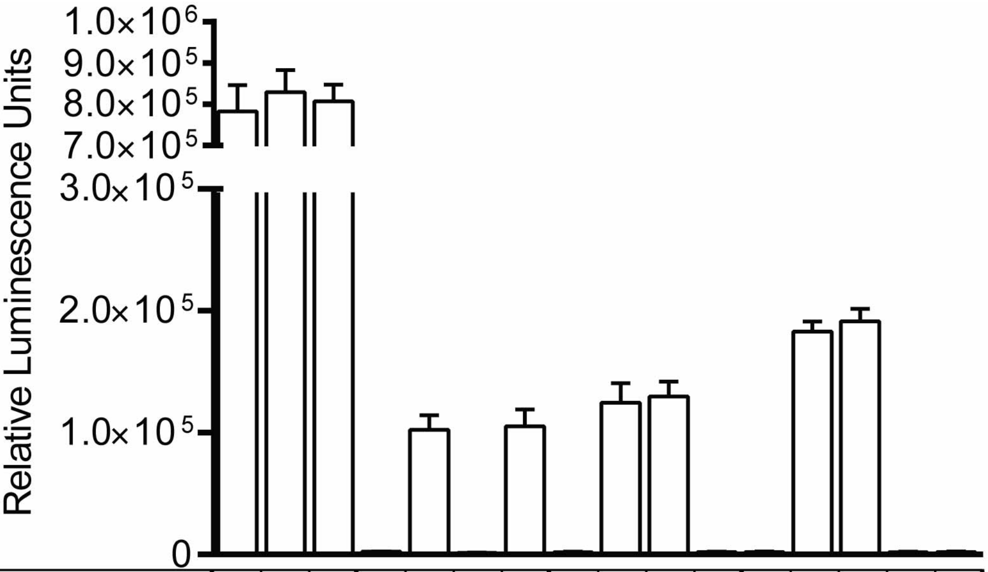

Nluc-AtCAX1	-	-	-	+	+	+	+	-	-	-	-	+	+	-	+	+
Nluc-AtCAX3	-	-	-	-	-	-	-	+	+	+	+	-	-	+	-	-
AtCAX1-Cluc	-	-	-	+	-	-	-	-	-	-	-	-	-	-	-	-
AtCAX3-Cluc	-	-	-	-	-	-	-	+	-	-	-	+	-	-	-	-
Cluc-AtCAX1-Cluc	-	-	-	-	+	+	+	-	-	-	-	-	-	+	-	-
Cluc-AtCAX3-Cluc	-	-	-	-	-	-	-	-	+	+	+	-	+	-	+	+
AtCAX1 amiRNA	-	+	-	-	-	+	-	-	-	+	-	-	-	-	+	-
AtCAX3 amiRNA	-	-	+	-	-	-	+	-	-	-	+	-	-	-	-	+
Renilla Luciferase	+	+	+	-	-	-	-	-	-	-	-	-	-	-	-	-

Supplemental Figure 7. Split luciferase assay protein-protein interaction assay determining CAX1 and CAX3 interactions in mesophyll protoplasts. Full-length CAX1 and CAX3 lacking a stop codon were recombined into split luciferase vectors with either the N-terminal (Nluc) or C-terminal (Cluc) half of luciferase fused to the N- or C-terminus of CAX1 or CAX3. amiRNAs specific for CAX1 or CAX3 were co-transfected to confirm the specificity of the interaction. Full length Renilla luciferase was used as a positive control.

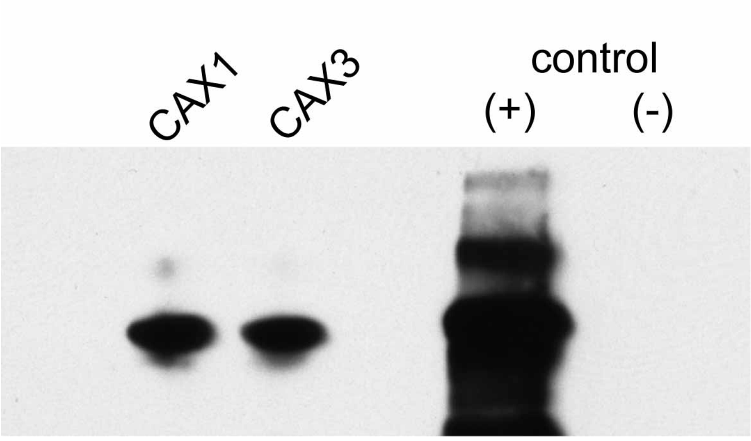

Supplementary Figure 8. Western blots showing relative levels of CAX1 and CAX3. Western blot analysis was performed on vacuolar-enriched protein extracted from C-terminally triple-HA-tagged full-length CAX1 and CAX3. Fifty micrograms of microsomal samples was separated by sodium dodecyl sulfate polyacrylamide gel electrophoresis, blotted, and subjected to Western blot analysis using a monoclonal antibody reactive to hemagglutinin.

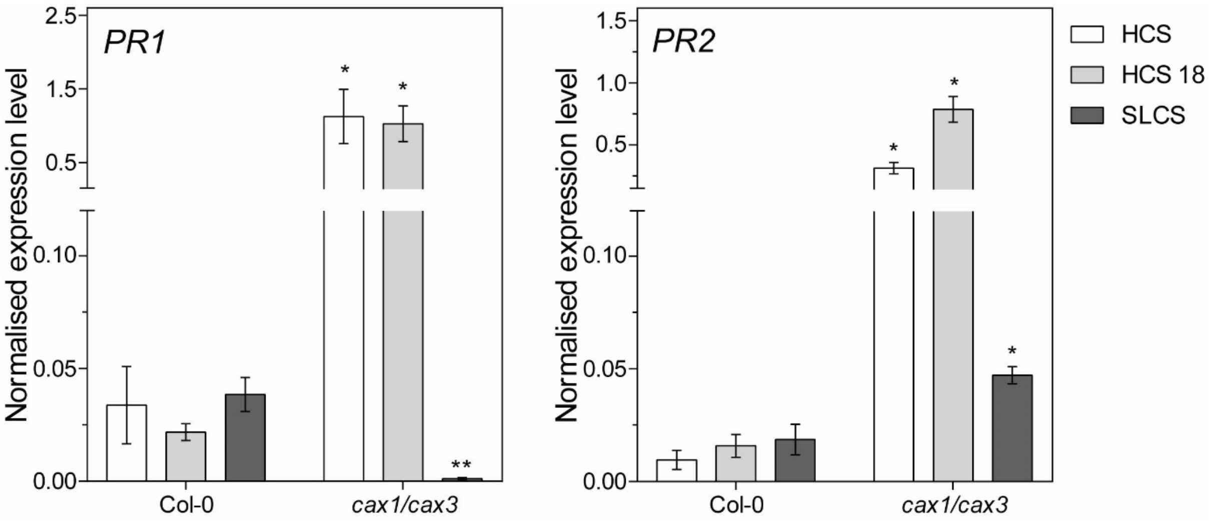

Supplemental Figure 9. Expression of *PR1* and *PR2* in 6-week old Col-0 and *cax1/cax3* shoots under SLCS and HCS treatment. Data represent Mean ± S.E.M, n =3. Expression level was normalised against *Actin2*, *EF1a*, Asterisks indicate significant difference from Col-0 control in the same treatments, as determined by *Student's t*-test (*$P < 0.05$, ** $P < 0.01$). HCS is for plants treated with 11 mM Ca for 1 week, HCS 18, is treatment for 18 hours.
